# Discovery of 1*H*-benzo[*d*]imidazole-(halogenated)Benzylidenebenzohydrazide Hybrids as Potential Multi-Kinase Inhibitors

**DOI:** 10.3390/ph17070839

**Published:** 2024-06-26

**Authors:** Tebyan O. Mirgany, Hanadi H. Asiri, A. F. M. Motiur Rahman, Mohammed M. Alanazi

**Affiliations:** Department of Pharmaceutical Chemistry, College of Pharmacy, King Saud University, Riyadh 11451, Saudi Arabia; tmirgany@ksu.edu.sa (T.O.M.); hasiri@ksu.edu.sa (H.H.A.)

**Keywords:** 1*H*-benzo[*d*]imidazole, tyrosine kinase inhibitor, multiple kinase inhibitor, cancer, apoptosis

## Abstract

In an effort to develop improved and effective targeted tyrosine kinase inhibitors (TKIs), a series of twelve novel compounds with the structural motif “(*E*)-4-(((1*H*-benzo[*d*]imidazol-2-yl)methyl)amino)-*N*′-(halogenated)benzylidenebenzohydrazide” were successfully synthesized in three steps, yielding high product yields (53–97%). Among this new class of compounds, **6c** and **6h-j** exhibited excellent cytotoxic effects against four different cancer cell lines, with half-maximal inhibitory concentration (IC_50_) values ranging from 7.82 to 21.48 μM. Notably, compounds **6h** and **6i** emerged as the most potent inhibitors, demonstrating significant activity against key kinases such as EGFR, HER2, and CDK2. Furthermore, compound **6h** displayed potent inhibitory activity against AURKC, while **6i** showed potent inhibitory effects against the mTOR enzyme, with excellent IC_50_ values comparable with well-established TKIs. The mechanistic study of lead compound **6i** revealed its ability to induce cell cycle arrest and apoptosis in HepG2 liver cancer cells. This was accompanied by upregulation of pro-apoptotic caspase-3 and Bax and downregulation of anti-apoptotic Bcl-2. Additionally, molecular docking studies indicated that the binding interactions of compounds **6h** and **6i** with the target enzymes give multiple interactions. These results underscore the ability of compound **6i** as a compelling lead candidate warranting further optimization and development as a potent multi-targeted kinase inhibitor, which could have significant implications for the treatment of various cancers. The detailed structural optimization, mechanism of action, and in vivo evaluation of this class of compounds warrant further investigation to assess their therapeutic potential.

## 1. Introduction

**Figure 1:**
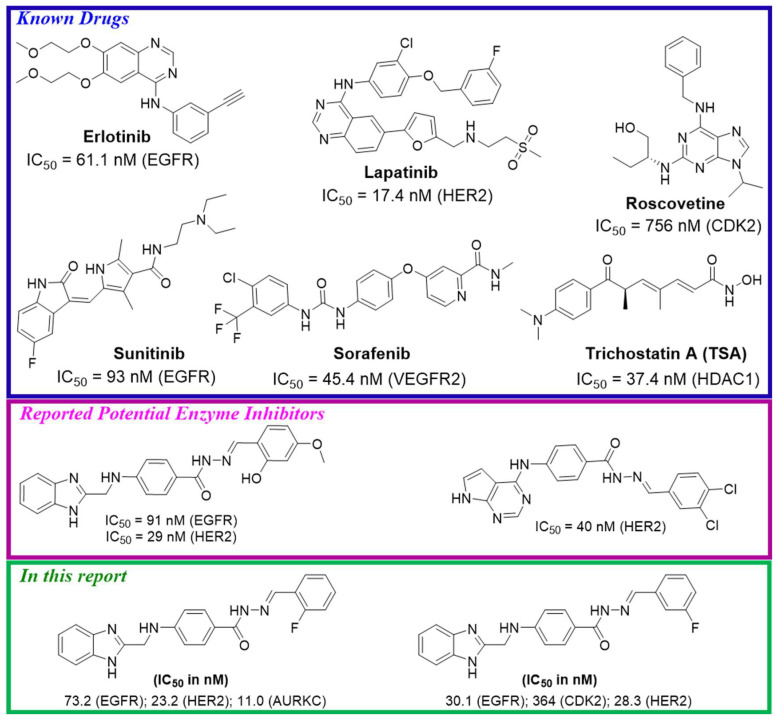
An example of some known TKIs [39], recently reported TKIs [40], and synthesized potential TKIs.

Cancer therapy continues to be a formidable task, demanding the discovery and development of effective treatments. Among the emerging classes of anticancer agents, halogenated TKIs have garnered significant attention owing to their unique chemical structures and enhanced pharmacological properties. Incorporating halogen atoms, such as fluorine, chlorine, bromine, or iodine, into the molecular backbone of TKIs offers a promising avenue to improve their therapeutic efficacy, selectivity, and potency [1]. The addition of halogen substituents in TKIs has been shown to exert a profound influence on their binding affinity to target kinases. Through their strategic placement, halogen atoms modulate the interactions between the TKIs and their kinase targets, potentially leading to enhanced therapeutic effects. These modifications can optimize the TKIs’ ability to bind to the kinase active site, resulting in increased potency and selectivity [1,2,3]. The halogenated TKI gefitinib, developed to precisely target the epidermal growth factor receptor (EGFR), has demonstrated its clinical utility in the care of non-small cell lung cancer (NSCLC) patients, leading to its adoption as a first-line standard of care therapy for advanced NSCLC cases harboring activating EGFR mutations. Furthermore, gefitinib offers advantages such as oral bioavailability and a comparatively lower toxicity profile, in contrast to conventional chemotherapy agents. Notably, it has shown promising outcomes in patients with EGFR mutations, which are frequently observed in Asian populations and allied with higher response rates to TKIs (Figure 1) [4,5,6,7,8]. Dasatinib, another halogenated TKI, exerts its therapeutic effects by targeting multiple tyrosine kinases, including BCR-ABL, SRC family kinases, and c-KIT. The incorporation of halogen substituents in dasatinib has been demonstrated to enhance its potency and selectivity, rendering it a valuable therapeutic option for various types of cancer. Notably, dasatinib has demonstrated efficacy in treating chronic myeloid leukemia (CML) and acute lymphoblastic leukemia (ALL), warranting its approval as a first-line or second-line therapy for these conditions. Additionally, dasatinib has demonstrated the capacity to overcome resistance to other TKIs, such as imatinib, which are commonly employed in the treatment of CML. However, it is crucial to recognize the potential adverse effects associated with dasatinib, which may include fluid retention, bleeding, and pulmonary arterial hypertension (PAH). Furthermore, the higher cost linked to the use of dasatinib is an important consideration [6]. Afatinib, a halogenated TKI with targets including EGFR, HER2, and HER4, has emerged as an effective therapy for NSCLC and head and neck cancer. Halogen substituents incorporated into afatinib have demonstrated the potential to enhance its potency and selectivity, thus improving its effectiveness in specific patient populations. Afatinib’s broad spectrum of activity against multiple members of the HER family of receptor tyrosine kinases positions it as a valuable treatment option for diverse cancer types. It has shown efficacy, particularly in patients with EGFR mutations, leading to its approval as a first-line therapy in this population. Additionally, afatinib has shown the capability of overwhelming resistance to other EGFR TKIs, including gefitinib and erlotinib. However, it is essential to consider potential adverse effects, such as diarrhea, rash, and mucositis, as well as the higher cost associated with afatinib [7,8]. Several halogenated TKIs have shown promising results in clinical trials and preclinical studies. Entrectinib, a halogenated TKI, has demonstrated efficacy in targeting ROS1 and NTRK fusions in solid tumors, including lung cancer and neuroblastoma [9]. Avitinib, another halogenated TKI, has shown potential in inhibiting the EGFR T790M mutation, which is allied with battling first-generation EGFR TKIs [10,11]. Saracatinib, a halogenated Src/Abl kinase inhibitor, has exhibited promising activity in solid tumors, particularly in pancreatic cancer [12,13]. Ponatinib, a halogenated TKI with potent activity against BCR-ABL and other tyrosine kinases, has been effective in treating chronic myeloid leukemia (CML) and Philadelphia chromosome-positive acute lymphoblastic leukemia (Ph+ ALL) [6,14] Foretinib, a halogenated TKI targeting MET, VEGFR, and other kinases, has shown efficacy in various solid tumors, including renal cell carcinoma and hepatocellular carcinoma [15]. Vandetanib, flumatinib, vemurafenib, sorafenib, cabozantinib, nilotinib, lapatinib, and selumetinib, all halogenated TKIs, have demonstrated efficacy in different cancer types by targeting specific kinases involved in tumor growth and progression [16,17,18,19,20,21,22,23,24]. These include VEGFR, BRAF, c-Met, RET, and HER2, among others. However, it is important to consider the potential adverse effects associated with these halogenated TKIs. These halogenated tyrosine kinase inhibitors may cause debilitating fatigue, cognitive deficits, and even life-threatening lung disease. Patients may also experience gastrointestinal issues, blood cell depletion, heart rhythm abnormalities, high blood pressure, painful skin, nail changes, and heightened photosensitivity. Vigilant monitoring and active management of these diverse adverse effects are crucial when prescribing these halogenated TKI therapies. Close monitoring and management of these side effects are crucial for ensuring patient safety and treatment effectiveness. Moreover, it is worth noting that some of these halogenated TKIs may have limitations, including high costs and limited accessibility in certain healthcare systems. The affordability and availability of these drugs are important factors to consider when evaluating their clinical utility and impact on patient care. However, halogenated TKIs have emerged as a promising class of anticancer agents, with multiple compounds demonstrating efficacy in various cancer types. While these drugs offer significant therapeutic potential, it is essential to carefully assess their adverse effects and limitations to optimize their clinical utility and ensure equitable access to effective treatments in cancer care. Further research and clinical investigations are warranted to elucidate the full potential of halogenated TKIs and their role in improving patient outcomes in oncology.

On the other hand, benzimidazole compounds have gained attention in drug development owing to their versatility and diverse applications. They exhibit antibacterial properties [25], potential as anti-tubercular agents [26], antifungal activities [27], and antiprotozoal effects [28]. Benzimidazole derivatives also show promise as antiviral agents [29] and as protein kinase inhibitors [30,31]. Studies have demonstrated the therapeutic action of benzimidazole-based compounds, such as nazartinib, in inhibiting EGFR and HER2 proteins [32,33,34,35]. Our previous research focused on hydrazone derivatives, which exhibited potent multi-kinase inhibition activity, including EGFR and HER2 [36,37,38,39]. To further explore their potential, we designed benzimidazole and (halogenated)benzylidene-benzohydrazide hybrids, namely, (*E*)-4-(((1*H*-benzo[d]imidazol-2-yl)methyl)amino)-*N*′-(halogenated)benzylidenebenzohydrazides, aiming for multi-kinase inhibitory activities. The synthesized compounds (**6a-l**) were evaluated for their in-vitro cytotoxicity against cancerous/normal cell lines, and their ability to inhibit multiple tyrosine kinases, investigated for their apoptosis-inducing ability, cell cycle-suppressing effects, drug-likeness properties, and in silico molecular docking, shed light on their selective mechanism of action and substantiated their potential as promising anticancer agents.

## 2. Results and Discussion

### 2.1. Chemistry

**Scheme 1:**
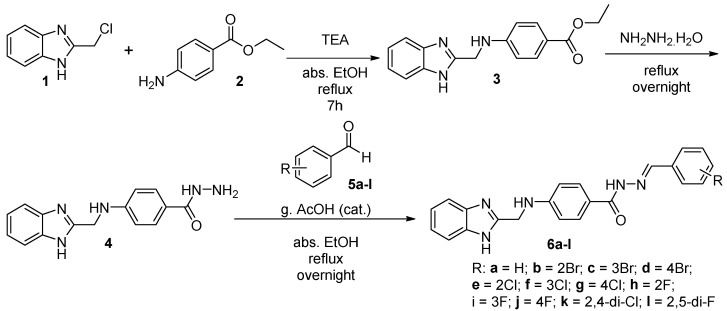
Synthesis of (*E*)-4-(((1*H*-benzo[*d*]imidazol-2-yl)methyl)amino)-*N*’-(substituted)benzylidenebenzohydrazide (**6a-l**).

The synthesis of (*E*)-4-(((1*H*-benzo[d]imidazol-2-yl)methyl)amino)-*N*′-(halogenated)benzylidenebenzohydrazide derivatives (**6a-l**) was carried out using a straightforward method, as described in Scheme 1, following the multi-step procedure adopted for our previously reported method [39]. The use of a previously reported synthetic methodology, along with the straightforward nature of the individual steps, allowed for the efficient preparation of the desired series of halogenated benzylidenebenzohydrazide derivatives (**6a-l**) bearing the benzo[*d*]imidazole moiety. The structural elucidation of the synthesized compounds was carried out using a comprehensive analytical approach, employing a suite of spectroscopic techniques. These included infrared (IR) spectroscopy, mass spectrometry (MS), and proton and carbon nuclear magnetic resonance (^1^H-NMR and ^13^C-NMR) analyses (please see the Supplementary Materials for the spectra). Additionally, we also reported the physical properties of the compounds **6a-l**, specifically their color and melting point ranges. The ^1^H-NMR spectroscopic analysis of the synthesized compounds **6a-l** revealed characteristic signals for the secondary aromatic amine protons located at the 4-position of the benzohydrazide moiety. These amine proton signals were observed in the 7.01 to 7.06 ppm region of the ^1^H-NMR spectra as triplets, with coupling constant values ranging from 5.7 to 6.1 Hz. The observed chemical shift and coupling pattern of these amine proton signals provided evidence that the aromatic amine functionality was directly bonded to an aliphatic (CH2) carbon, specifically the methylene group derived from the condensation of compounds **1** and **2** during the synthetic sequence. These ^1^H-NMR data thus confirmed the successful formation of the desired compound **6a-l** structures, with the secondary aromatic amine protons serving as a characteristic spectroscopic signature for the benzohydrazide moiety within the target molecules. The ^1^H-NMR spectra of the synthesized compounds **6a-l** also exhibited characteristic signals for the benzylidene protons. These benzylidene proton signals were observed in the 8.35 to 8.38 ppm region of the ^1^H-NMR spectra, except for the compounds with substituents at the 2-position of the benzene ring. Specifically, compound **6b**, which contained a bromo substituent at the 2-position, displayed a downfield shift of 0.38 ppm compared with the non-substituted compound **6a**. Similarly, the 2-chloro substituted compound **6e** exhibited a 0.43 ppm downfield shift, while the 2-fluoro substituted compound **6h** showed a 0.27 ppm downfield shift relative to **6a**. Furthermore, the compounds with 2,4-dihalogen substitution, **6k** (2,4-dichloro) and **6l** (2,5-difluoro), also demonstrated downfield shifts of 0.38 ppm and 0.22 ppm, respectively, compared with the unsubstituted analog **6a**. These observed downfield shifts in the benzylidene proton signals for the 2-substituted and 2,4-disubstituted compounds can be attributed to the electronic effects of the halogen substituents, which influenced the chemical environment and deshielding of the benzylidene protons. 

The structures of the synthesized compounds **6a-l** were further proved by the observation of distinctive IR spectral signatures, such as the sharp peaks observed in the range of 1604–1613 cm^−1^, which were characteristic of the benzylidene hydrazone (-N=CH-) functional group present in all the compounds. N-H stretching vibration bonds of amide -NH (-CO-NH-) appeared between 3044 and 3464 cm^−1^ and around 1502–1568 cm^−1^. Carbonyl (C=O) vibrational bonds appeared at around 1642–1656 cm^−1^. Furthermore, each compound displayed distinct IR spectral features. Notably, the bromo-substituted analogs (**6b-d**) exhibited characteristic stretching bands in the range of 741–748 cm^−1^. In contrast, the chloro- and fluoro-substituted compounds (**6e-l**) showed distinctive stretching bands between 504 and 758 cm^−1^. Finally, the structures of compounds **6e-l** were further confirmed through their mass spectral analysis data.

### 2.2. Biological Evaluation

#### 2.2.1. In Vitro Cytotoxicity

The cytotoxic activities of compounds **6a-l** were assessed using a standard MTT method, which was performed across three different cancer cell line models, namely human colon cancer (HCT-116), hepatocellular carcinoma (HepG2), mammary gland cancer (MCF-7), and normal (WI-38) cell lines. The results presented in Table 1 show that the cytotoxicity of the tested compounds was quantified by the concentration required to induce 50% inhibition of cancer cell viability. The cytotoxicity results provide a comprehensive evaluation of the synthesized compounds **6a-6l** in comparison with the standard drugs sorafenib, doxorubicin, and sunitinib. Among the synthesized compounds, **6c** (3-Br substituted) and **6i** (3-F substituted) exhibited the most potent cytotoxic activity, with IC_50_ values ranging from 7.82 to 10.21 μM across the tested cancer cell lines. These values are comparable to the standard drugs, which have IC_50_ values between 4.17 and 24.06 μM. Compounds **6h** (2-F), **6j** (4-F), and **6d** (4-Br) also demonstrated relatively strong cytotoxicity, with IC_50_ values generally below **30** μM in the cancer cell lines. The remaining compounds, including **6a**, **6b**, **6e**, **6f**, **6g**, **6k**, and **6l**, exhibited moderate to weaker cytotoxic activity, with IC_50_ values mostly above 20 μM. When considering the selectivity toward cancer cells over the normal WI-38 fibroblast cell line, compounds **6c**, **6i**, and **6j** stood out, showing significantly lower IC_50_ values in the cancer cell lines compared with the WI-38 cells, indicating a higher degree of selectivity. Other compounds, such as **6h** and **6d**, also exhibited relatively better selectivity toward the cancer cell lines. The structure–activity relationship analysis suggested that the presence of bromo (Br) or fluoro (F) substituents at the 3-position of the phenyl ring is favorable for potent and selective cytotoxic activity, as observed with compounds **6c** and **6i**. Substitution at the 4-position, as in the case of compounds **6d**, **6g**, and **6j**, also seems beneficial, with fluoro (**6j**) showing the best results. In contrast, the disubstituted compounds **6k** and **6l** generally exhibited lower cytotoxic potency and selectivity compared with their monosubstituted counterparts. Overall, the cytotoxicity data provide valuable insights into the structure–activity relationships of the synthesized compounds and can guide further optimization efforts to develop potential anticancer agents with improved potency and selectivity.

The Selectivity Index (SI) values shown in Table 1 provide important information about the selectivity of the synthesized compounds (**6a-l**) against the various cancer cell lines tested. The SI values represent the ratio of the IC_50_ values for the normal WI-38 cell line compared with the respective cancer cell lines, with higher SI values indicating greater selectivity for the cancer cells over normal cells. Compounds **6c**, **6h**, and **6i** exhibited the highest SI values against the SI cancer cell line, with SI values of 4.20–4.72 (HCT-116), 5.41–7.19 (HepG2), and 4.85–6.16 (MCF-7), respectively. The SI values for the standard drugs were 1.16–1.95 (sorafenib), 1.28–1.61 (doxorubicin), and 2.31–3.11 (sunitinib), except 6.64 against the HepG2 cell line for sunitinib 6.64, respectively. This suggests these compounds (**6c**, **6h**, **6i**) demonstrated the greatest selectivity for the SI cancer cells compared with the normal WI-38 cells. 

#### 2.2.2. In Vitro Protein Kinase Inhibition Assays

Based on the cytotoxicity analysis of the synthesized compounds (**6a-l**), four compounds (**6c**, **6h-j**) were chosen for further enzymatic activity assessment against a range of kinase enzymes, including EGFR, Her2, VEGFR2, CDK2, AURKC, HDAC1, and mTOR (Table 2). To benchmark the activity of these compounds, several well-known kinase inhibitors were used as reference standards for the tested kinases, such as erlotinib for EGFR, lapatinib for Her2, sorafenib for VEGFR2, roscovetine for CDK2, TSA for AURKC and HDAC1, and rapamycin for mTOR. The synthesized compounds **6c** and **6h-j** demonstrated varying degrees of potent inhibition across the different protein kinases tested. For instance, compound **6h** exhibited very high potency against EGFR (IC_50_ = 73.2 nM), which is almost 1-fold higher than the standard erlotinib (IC_50_ = 61.1 nM), and against Her2 (IC_50_ = 23.2 nM), which is also almost 1-fold higher than lapatinib (IC_50_ = 17.4 nM). Additionally, **6h** showed high potency against CDK2 (IC_50_ = 284 nM), which is 2.5-fold higher than roscovetine (IC_50_ = 756 nM), and very high potency against AURKC (IC_50_ = 11 nM), which is 3-fold higher than the standard TSA (IC_50_ = 30.4 nM). Compound **6i** showed excellent potency against EGFR (IC_50_ = 30.1 nM), which is almost 2-fold higher than the standard erlotinib, and against Her2 (IC_50_ = 28.3 nM), which is almost 1-fold higher than lapatinib. Additionally, **6i** exhibited high potency against CDK2 (IC_50_ = 364 nM), which is 2-fold higher than roscovetine, and against mTOR (IC_50_ = 152 nM), which is 1-fold higher than the standard rapamycin (IC_50_ = 208 nM). In contrast, compound **6c** gave disappointing results compared with the expectations based on the cytotoxicity against various cancer cell lines. Additionally, compound **6j** showed very little inhibitory activity against the tested kinases. Compared with the standard drugs, the synthesized compounds **6h** and **6i** exhibited superior potency across multiple kinase targets. The low IC_50_ values for the synthesized compounds indicate their high binding affinity and inhibitory activity toward these clinically relevant protein kinases, suggesting their potential to be developed as potent and selective anticancer agents targeting multiple dysregulated signaling pathways in cancer. These findings underscore the promising potential of compounds **6h** and **6i** as candidates for further development as kinase inhibitors.

#### 2.2.3. Cell Cycle Analysis

**Figure 2:**
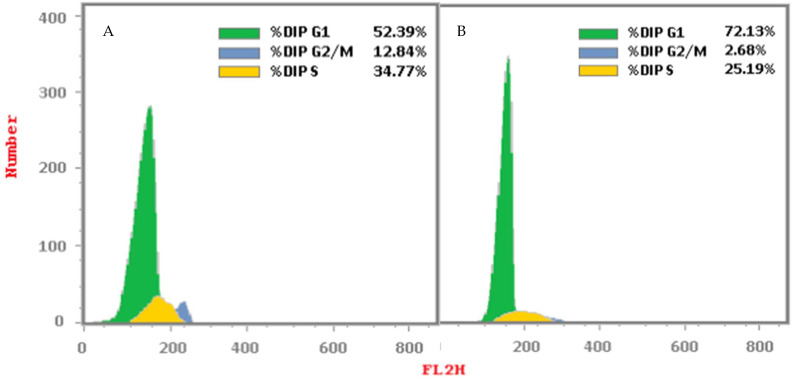
Cell cycle phases of HepG2 cells following treatment with compound **6i**: (**A**) Control HepG2 cell lines; (**B**) HepG2 cells treated with compound **6i**.

The cytotoxicity assay, selectivity index (SI) values, and enzyme inhibitory activities of the synthesized compounds **6a-l** were evaluated, and based on these results, compound **6i** was selected for further investigation to assess its impact on cell cycle progression in HepG2 cells. To examine the effect of compound **6i** on the cell cycle, HepG2 cells were treated with the compound at its IC_50_ concentration for 24 h. Following the treatment, the cells were stained with propidium iodide and analyzed using flow cytometry to determine the cell cycle phase distribution. The cell cycle analysis results, presented in Table 3 and Figure 2, revealed that treatment with compound **6i** elicited a distinct cell cycle arrest effect. Specifically, the data showed that the DNA content of cells treated with **6i** increased in the G1 phase while decreasing in the S and G2/M phases. This indicates that compound **6i** induced a significant cell cycle arrest at the G1 phase. The quantitative data further substantiated this observation. The percentage of cells in the G0-G1 phase increased from 52.39% in the untreated control to 72.13% upon treatment with compound **6i**. Conversely, the proportion of cells in the S phase decreased from 34.77% to 25.19%, and the percentage of cells in the G2/M phase declined from 12.84% to 2.68%. These findings suggest that treatment with compound **6i** substantially altered the cell cycle distribution of the HepG2 cancer cells. It led to a marked accumulation of cells in the G1 phase, accompanied by a concomitant reduction in the proportion of cells in the S and G2/M phases. The cell cycle arrest effect observed with compound **6i** may be a key mechanistic aspect contributing to its anticancer potential. The observed cell cycle arrest at the G1 phase is a common mechanism of action for many anti-cancer agents, as it can prevent cell division and proliferation, ultimately leading to cell cycle arrest and potentially inducing apoptosis or other forms of cell death. These results, along with the previously reported cytotoxicity and kinase inhibitory activities of compound **6i**, further highlight its potential as a promising anticancer candidate that warrants further investigation and optimization for targeted cancer therapy.

The cell cycle distribution index (CDI) is a metric that quantifies the rate of cell proliferation, calculated as CDI = (G2/M + S)/(G0 − G1), where the values represent the percentages of cells in each phase. In this study, the CDI values for the control and compound **6i**-treated HepG2 cells were 0.91 and 0.39, respectively. A decrease in CDI indicates a reduced rate of cell proliferation, suggesting cell cycle arrest. Compound **6i** was further evaluated for apoptosis analysis in HepG2 cell lines.

#### 2.2.4. Apoptosis Analysis

##### Annexin-V/Propidium Iodide (PI) Staining Assay

**Figure 3:**
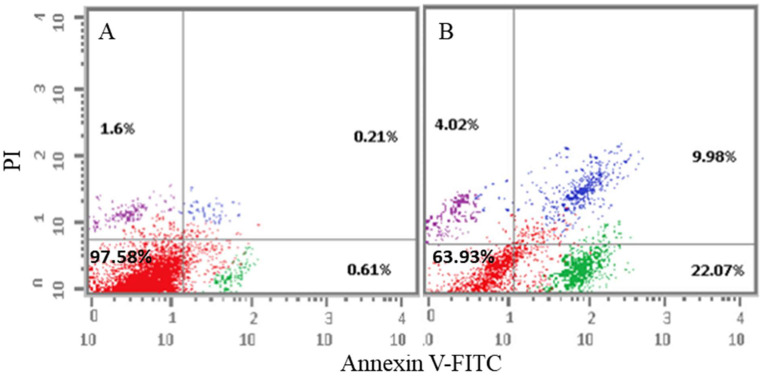
Apoptotic cell distributions: (**A**) Control HepG2 cells; (**B**) HepG2 cells treated with compound **6i**. The colors red, green, blue and purple are representing alive cells, early apoptosis, late apoptosis and necrosis, respectively.

To investigate the mode of cell death induced by the synthesized compound **6i**, flow cytometry analysis was performed. HepG2 cancer cells were treated with compound **6i** at their respective IC_50_ concentrations and incubated for 24 h. Following the treatment, the cells were double-stained with Annexin V and propidium iodide (PI) to assess the levels of apoptosis and necrosis. The data presented in Table 4 reveal a significant decline in the percentage of viable cells in the compound **6i**-treated group (63.93%) compared with the untreated control group (97.58%). In the early stages of the cell death process, the percentage of cells undergoing apoptosis was 0.61% in the control group, whereas it increased to 22.07% in the compound **6i**-treated group. Additionally, in the late stages of apoptosis, the percentage of apoptotic cells rose from 0.21% in the control group to 9.98% in the compound **6i**-treated group. These findings suggest that the antiproliferative effects of compound **6i** against HepG2 cancer cells are at least partially mediated through the induction of apoptosis, particularly during the early stages of the cell death process. The flow cytometry data indicate that treatment with compound **6i** leads to a substantial increase in the proportion of HepG2 cells undergoing both early and late stages of apoptosis. The observed increase in both early and late apoptotic cell populations upon treatment with compound **6i** indicates that this compound may trigger the apoptotic signaling cascades, leading to programmed cell death. Additionally, the data show a slight increase in the percentage of necrotic cells from 1.6% in the control group to 4.02% in the compound **6i**-treated group, suggesting that the compound may also induce some degree of necrosis in HepG2 cells. Taken together, these results highlight the pro-apoptotic and potentially cytotoxic effects of compound **6i** on HepG2 cells, which could have important implications for its further development as a potential anticancer agent. The apoptosis levels in the control HepG2 cell lines and the cells treated with compound **6i** are shown in the graphical representation provided in Figure 3.

##### Determination of Apoptotic Protein Levels

To further elucidate the mechanism by which compound **6i** induces cell death, the expression levels of key apoptotic proteins were assessed in the HepG2 cell line. As shown in Table 5, treatment with compound **6i** resulted in significant changes in the levels of critical proteins involved in the apoptotic signaling cascade in HepG2 cells. Specifically, the levels of the pro-apoptotic proteins caspase-3 and Bax were markedly higher in the compound **6i**-treated group compared with the control. Caspase-3 levels were approximately 3.9-fold higher, while Bax levels were 7.22-fold higher in the treated cells. In contrast, the level of the anti-apoptotic protein Bcl-2 was 7.5-fold lower in the compound **6i**-treated group compared with the control. These findings strongly suggest that the induction of apoptosis by compound **6i** in HepG2 cells is mediated, at least in part, by the upregulation of the pro-apoptotic proteins caspase-3 and Bax, coupled with the downregulation of the anti-apoptotic protein Bcl-2. The magnitude of the changes observed in the levels of these key apoptotic regulators upon treatment with compound **6i** was comparable to, or even exceeded, the effects seen with the known apoptosis-inducing agent staurosporine. These results further substantiate the pro-apoptotic mechanism of action of compound **6i** and highlight its potential as a promising candidate for the development of novel therapies targeting diseases involving dysregulated cell death, such as cancer. Additional studies are warranted to elucidate the detailed molecular mechanisms underlying the apoptosis-inducing effects of compound **6i** and to assess its therapeutic potential in relevant in vivo models.

### 2.3. In Silico Studies

#### Molecular Docking

In order to predict the potential binding interactions between compounds **6h** and **6i** and the investigated protein kinase enzymes, compound **6h** was docked into the active site of Her2 (PDB: 7PCD) and AURKC (PDB: 6GR8), and **6i** was docked into the active side of EGFR (PDB: 4HJO) and Her2 (PDB: 7PCD), respectively. 

**Figure 4:**
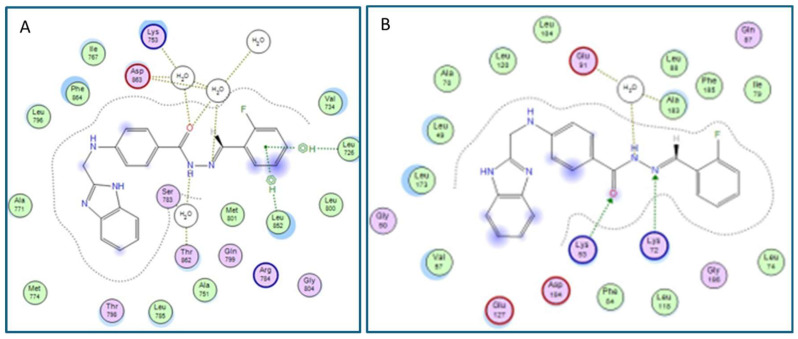
Docking study of compound **6h** showing different types of binding interaction with the key amino acid residues: (**A**) **6h** with Her2; and (**B**) **6h** with AURKC kinase enzymes.

Based on the docking data of compound **6h** with the HER2 kinase enzyme (PDB: 7PCD), the hydrogen bond network with Lys753, Asp863, and Thr862 through water molecules stabilized the compound inside the active site of the target enzyme (Figure 4A). Additionally, binding interactions between the benzylidene benzene moiety of compound **6h** and the amino acid residues Leu726 and Leu852 were observed, involving H-π bonds (Figure 4A). However, when compound **6h** was co-crystallized with the AURKC kinase enzyme (PDB: 6GR8), three hydrogen bonds were formed between the key amino acid residues and the hydrazide moiety, facilitating the fitting of the compound within the active site (Figure 4B). Specifically, one hydrogen bond was between the side chain of Lys35 and the carbonyl group, the second hydrogen bond was between Lys72 and a nitrogen atom, and the third hydrogen bond was part of a network involving Glu91 and Ala183, mediated by a water molecule (Figure 4B).

**Figure 5:**
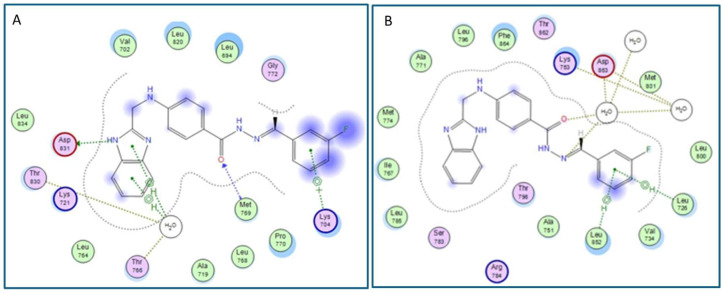
Docking study of compound **6i** showing different types of binding interaction with the key amino acid residues: (**A**) compound **6i** with EGFR; and (**B**) compound **6i** with Her2 kinase enzymes.

The docking study results of compound **6i** with the EGFR (PDB: 4HJO) and the active site of HER2 kinase enzyme (PDB: 7PCD) are depicted in Figure 5. For the EGFR kinase enzyme, the benzimidazole moiety of compound **6i** formed a hydrogen bonding network, mediated by water molecules, with side chains of Thr766 and Thr830 (Figure 5A). Additionally, the nitrogen atom of the imidazole ring participated in a hydrogen bond with the key amino acid residue Asp831, while another important hydrogen bond was formed between the backbone of Met769 and the carbonyl oxygen of the compound, facilitating its fitting within the active site. Furthermore, the presence of a fluorine atom in the meta position of the benzene ring enhanced its interaction with Lys704 through the formation of a cation-π bond compared with other derivatives (Figure 5A). For the Her2 active site, compound **6i** made a hydrogen bond network with Lys753, Asp863, and Met501, stabilized by water molecules inside the active site (Figure 5B). Additionally, binding interactions between the benzylidene benzene moiety of compound **6i** and the amino acid residues Leu726 and Leu852 were also observed, similar to the interactions seen for compound **6h**. 

## 3. Materials and Methods

### 3.1. General 

All reagents and solvents used in the experiments were of commercial grade and employed without further purification. Barnstead electrothermal digital melting point apparatus (model IA9100, BIBBY scientific limited, Staffordshire, UK) was used to determine the melting points. A Jasco FT/IR-6600 spectrometer (Tokyo, Japan) was used for recording IR data. Bruker 700 MHz NMR spectrometry (Zurich, Switzerland) was used to obtain the NMR data. Mass spectra were taken using an Agilent 6320 ion trap mass spectrometer equipped with an ESI ion source (Agilent Technologies, Palo Alto, CA, USA).

### 3.2. Chemistry 

#### 3.2.1. Synthesis of Ethyl 4-(((1*H*-benzo[d]imidazol-2-yl)methyl)amino)benzoate (**3**)

Ethyl 4-(((1*H*-benzo[*d*]imidazol-2-yl)methyl)amino)benzoate (**3**) was prepared using a previously reported method [39]. White powder (65%), mp. 255 °C (lit. [39] mp. = 255 °C).

#### 3.2.2. Synthesis of 4-(((1*H*-benzo[*d*]imidazol-2-yl)methyl)amino)benzohydrazide (**4**)

Additionally, 4-(((1*H*-Benzo[*d*]imidazol-2-yl)methyl)amino)benzohydrazide (**4**) was prepared using our previously reported method [39]. White powder (80%), p. 240 °C (lit. [39] mp. = 240 °C). 

### 3.3. General Procedure for the Preparation of (E)-4-(((1H-benzo[d]imidazol-2-yl)methyl)amino)-N′-(substitutedbenzylidene)benzohydrazide (**6a-l**)

An equimolar mixture of hydrazide **4** and halogen-substituted benzaldehyde (**5**) was reacted to obtain the desired 1*H*-benzo[*d*]imidazole-(halogenated)benzylidenebenzohydrazide (**6a-l)** following the reported procedure [39,40,42]. 

#### 3.3.1. (*E*)-4-(((1*H*-benzo[d]imidazol-2-yl)methyl)amino)-*N*′-benzylidenebenzohydrazide (**6a**)

White solid (53.43%), mp. 278 °C (lit. [39] mp. = 278 °C; lit [43] mp. = 246 °C), CAS registry number 76321-88-5.

#### 3.3.2. (*E*)-4-(((1*H*-benzo[d]imidazol-2-yl)methyl)amino)-*N*′-(2-bromobenzylidene)benzohydrazide (**6b**)

White solid (105 mg, 0.234 mmol, 66%), mp. 284 °C. FT-IR (KBr): ν (cm^−1^) = 3437, 3174, 3135, 3044, 2976, 2829, 1926, 1893, 1649, 1604, 1558, 1522, 1431, 1350, 1294, 1268, 1187,1057, 1014, 832, 761, 739, 689, 647, and 608 cm^−1^. ^1^H-NMR (700 MHz, DMSO-*d*_6_), δ ^1^H NMR (700 MHz, DMSO), δ 12.35 (s, 1H), 11.76 (s, 1H), 8.75 (s, 1H), 7.99 (s, 1H), 7.75 (d, *J* = 8.3 Hz, 2H), 7.69 (d, *J* = 8.2 Hz, 1H), 7.58 (s, 1H), 7.46 (t, *J* = 7.7 Hz, 2H), 7.35 (t, *J* = 7.6 Hz, 1H), 7.15 (s, 2H), 7.04 (t, *J* = 6.1 Hz, 1H), 6.74 (d, *J* = 8.3 Hz, 2H), and 4.59 (d, *J* = 5.8 Hz, 2H) ppm. ^13^C-NMR (176 MHz, DMSO-*d*_6_), δ 163.54, 153.45, 152.11, 144.83, 143.78, 134.90, 133.98, 133.68, 131.93, 129.87, 128.62, 127.67, 123.87, 122.38, 121.66, 120.71, 118.98, 111.98, 111.80, and 41.85 ppm. Mass (ESI): *m*/*z* 448 [^79^(Br)M+H]^+^, 450 [^81^(Br)M+H]^+^.

#### 3.3.3. (*E*)-4-(((1*H*-benzo[d]imidazol-2-yl)methyl)amino)-*N*′-(3-bromobenzylidene)benzohydrazide (**6c**)

White solid (97 mg, 0.216 mmol, 61%), mp. 278 °C. FT-IR (KBr): ν (cm^−1^) = 3388, 3096, 2963, 2907, 2845, 2790, 1963, 1656, 1606, 1522, 1438, 1255, 1073, 1021, 895, 822, 741,692, 640, and 478 cm^−1^. ^1^H-NMR (700 MHz, DMSO-*d*_6_), δ ^1^H NMR (700 MHz, DMSO), δ 12.35 (s, 1H), 11.62 (s, 1H), 8.35 (s, 1H), 7.89 (s, 1H), 7.73 (d, *J* = 8.3 Hz, 2H), 7.68 (d, *J* = 8.0 Hz, 1H), 7.60 (d, *J* = 8.2 Hz, 1H), 7.57 (s, 1H), 7.45 (s, 1H), 7.41 (t, *J* = 8.0 Hz, 1H), 7.15 (s, 2H), 7.04 (t, *J* = 6.1 Hz, 1H), 6.73 (d, *J* = 8.5 Hz, 2H), and 4.58 (d, *J* = 5.7 Hz, 2H) ppm. ^13^C-NMR (176 MHz, DMSO-*d*_6_), δ 163.46, 153.35, 151.97, 144.67, 143.67, 137.64, 134.79, 132.65, 131.45, 129.73, 129.29, 126.49, 122.62, 122.27, 121.56, 120.68, 118.87, 111.87, 111.70, and 41.75 ppm. Mass (ESI): *m*/*z* 448 [^79^(Br)M+H]^+^, 450 [^81^(Br)M+H]^+^.

#### 3.3.4. (*E*)-4-(((1*H*-benzo[d]imidazol-2-yl)methyl)amino)-*N*′-(4-bromobenzylidene)benzohydrazide (**6d**)

White solid (124 mg, 0.276 mmol, 63%), mp. 287 °C (lit. [43] mp. = 230 °C), CAS registry number 76321-87-4. FT-IR (KBr): ν (cm^−1^) =3389, 3060, 2957, 2906, 2796, 1655, 1610, 1568, 1526, 1487, 1455, 1438, 1357, 1335, 1296, 1276, 1260, 1212, 1190, 1150, 1132, 1068, 1026, 1009, 956, 934, 825, 748, 695, 638, 515, and 503 cm^−1^. ^1^H-NMR (700 MHz, DMSO-*d*_6_), δ 12.35 (s, 1H), 11.56 (s, 1H), 8.36 (s, 1H), 7.72 (d, *J* = 8.6 Hz, 2H), 7.64 (s, 4H), 7.58 (s, 1H), 7.45 (s, 1H), 7.15 (s, 2H), 7.03 (s, 1H), 6.73 (d, *J* = 8.7 Hz, 2H), and 4.58 (d, *J* = 5.8 Hz, 2H) ppm. ^13^C-NMR (176 MHz, DMSO-*d*_6_), δ 163.40, 153.37, 151.93, 145.29, 143.68, 134.80, 134.43, 132.27, 129.70, 129.17, 123.31, 122.29, 121.56, 120.79, 118.88, 111.88, 111.71, and 41.76 ppm. Mass (ESI): *m*/*z* 448 [^79^(Br)M+H]^+^, 450 [^81^(Br)M+H]^+^. 

#### 3.3.5. (*E*)-4-(((1*H*-benzo[d]imidazol-2-yl)methyl)amino)-*N*′-(2-chlorobenzylidene)benzohydrazide (**6e**)

White solid (140 mg, 0.346 mmol, 97%), mp. 289 °C. FT-IR (KBr): ν (cm^−1^) = 1656, 1606, 1522, 1431, 1360, 1330, 1298, 1258, 1187, 1148, 1132, 1050, 1024, 936, 826, 744, 713, 692, 640, and 498 cm^−1^. ^1^H-NMR (700 MHz, DMSO-*d*_6_), δ ^1^H NMR (700 MHz, DMSO), δ 12.35 (s, 1H), 11.73 (s, 1H), 8.80 (s, 1H), 8.00 (s, 1H), 7.74 (d, *J* = 8.8 Hz, 2H), 7.58 (d, *J* = 7.6 Hz, 1H), 7.52 (d, *J* = 4.4 Hz, 1H), 7.43 (t, *J* = 8.4 Hz, 3H), 7.15 (s, 2H), 7.04 (t, *J* = 6.1 Hz, 1H), 6.74 (d, *J* = 8.7 Hz, 2H), and 4.58 (d, *J* = 5.7 Hz, 2H) ppm. ^13^C-NMR (176 MHz, DMSO-*d*_6_), δ 163.39, 158.77, 153.34, 152.01, 143.68, 142.37, 134.79, 133.37, 132.38, 131.57, 130.35, 129.75, 128.04, 127.18, 122.29, 121.55, 120.60, 118.87, 111.88, 111.70, and 41.74 ppm. Mass (ESI): *m*/*z* 404 [^35^(Cl)M+H]^+^, 406 [^37^(Cl)M+H]^+^. 

#### 3.3.6. (*E*)-4-(((1*H*-benzo[d]imidazol-2-yl)methyl)amino)-*N*′-(3-chlorobenzylidene)benzohydrazide (**6f**)

White solid (136 mg, 0.336 mmol, 94%), mp. 271 °C. FT-IR (KBr): ν (cm^−1^) = 3398, 3057, 3025, 2871, 2836, 1609, 1525, 1473, 1434, 1334, 1281, 1184, 1132, 1024, 920, 822, 739, 696, 621, and 556 cm^−1^. ^1^H-NMR (700 MHz, DMSO-*d*_6_), δ ^1^H NMR (700 MHz, DMSO), δ 12.35 (s, 1H), 11.62 (s, 1H), 8.37 (s, 1H), 7.74 (s, 1H), 7.73 (d, *J* = 8.8 Hz, 2H), 7.65 (s, 1H), 7.58 (d, *J* = 7.6 Hz, 1H), 7.47 (d, *J* = 5.2 Hz, 2H), 7.44 (d, *J* = 7.5 Hz, 1H), 7.18–7.12 (m, 2H), 7.04 (t, *J* = 5.8 Hz, 1H), 6.74 (d, *J* = 8.8 Hz, 2H), and 4.58 (d, *J* = 5.8 Hz, 2H) ppm. ^13^C-NMR (176 MHz, DMSO-*d*_6_), δ 163.49, 153.35, 151.97, 144.78, 137.41, 134.80, 134.09, 131.17, 129.77, 126.46, 126.06, 122.29, 121.55, 120.70, 118.87, 111.87, 111.70, and 41.76 ppm. Mass (ESI): *m*/*z* 404 [^35^(Cl)M+H]^+^, 406 [^37^(Cl)M+H]^+^.

#### 3.3.7. (*E*)-4-(((1*H*-benzo[d]imidazol-2-yl)methyl)amino)-*N*′-(4-chlorobenzylidene)benzohydrazide (**6g**)

White solid (100 mg, 0.247 mmol, 69%), mp. 290 °C (lit. [43] mp. = 238 °C), CAS registry number 76321-91-0. FT-IR (KBr): ν (cm^−1^) = 3385, 3057, 2952, 2911, 2787, 2514, 1656, 1606, 1522, 1434, 1334, 1294, 1255, 1210, 1184, 1086, 1026, 934, 822, 741, 637, 504, and 436 cm^−1^. ^1^H-NMR (700 MHz, DMSO-*d*_6_), δ 12.35 (s, 1H), 11.55 (s, 1H), 8.38 (s, 1H), 7.72 (d, *J* = 8.5 Hz, 4H), 7.57 (s, 1H), 7.51 (d, *J* = 8.3 Hz, 2H), 7.44 (s, 1H), 7.14 (s, 2H), 7.02 (t, *J* = 5.8 Hz, 1H), 6.73 (d, *J* = 8.6 Hz, 2H), and 4.58 (d, *J* = 5.8 Hz, 2H) ppm. ^13^C-NMR (176 MHz, DMSO-*d*_6_), δ 163.38, 153.37, 151.92, 145.20, 143.69, 134.80, 134.54, 134.09, 129.69, 129.36, 128.92, 122.28, 121.55, 120.79, 118.87, 111.87, 111.70, and 41.76 ppm. Mass (ESI): *m*/*z* 404 [^35^(Cl)M+H]^+^, 406 [^37^(Cl)M+H]^+^.

#### 3.3.8. (*E*)-4-(((1*H*-benzo[d]imidazol-2-yl)methyl)amino)-*N*′-(2-fluorobenzylidene)benzohydrazide (**6h**)

White solid (100 mg, 0.258 mmol, 72%), mp. 289 °C. FT-IR (KBr): ν (cm^−1^) = 3388, 3096, 2963, 2907, 2845, 2790, 1963, 1655, 1606, 1521, 1437, 1255, 1073, 1021, 894, 822, 741, 692, 640, and 478 cm^−1^. ^1^H-NMR (700 MHz, DMSO-*d*_6_), δ 12.35 (s, 1H), 11.62 (s, 1H), 8.64 (s, 1H), 7.92 (s, 1H), 7.73 (d, *J* = 8.7 Hz, 2H), 7.58 (d, *J* = 8.0 Hz, 1H), 7.47 (d, *J* = 6.1 Hz, 1H), 7.45 (d, *J* = 7.6 Hz, 1H), 7.29 (t, *J* = 8.5 Hz, 2H), 7.15 (s, 2H), 7.04 (s, 1H), 6.74 (d, *J* = 8.7 Hz, 2H), and 4.58 (d, *J* = 5.8 Hz, 2H) ppm. ^13^C-NMR (176 MHz, DMSO-*d*_6_), δ 163.37, 160.61 (^1^*J*_C-F_ = 250 Hz), 153.36, 151.97, 143.67, 139.15, 134.79, 132.03 (^3^*J*_C-F_ = 8.4 Hz), 129.71, 126.63 (^4^*J*_C-F_ = 2.8 Hz), 125.37 (^3^*J*_C-F_ = 3Hz), 122.66 (^2^*J*_C-F_ = 9.8 Hz), 122.30, 121.56, 120.66, 118.88, 116.43 (^2^*J*_C-F_ = 20.8 Hz), 111.90, 111.70, and 41.74 ppm. Mass (ESI): *m*/*z* 388 [(^18^F)M+H]^+^; 389 [(^19^F)M+H]^+^.

#### 3.3.9. (*E*)-4-(((1*H*-benzo[d]imidazol-2-yl)methyl)amino)-*N*′-(3-fluorobenzylidene)benzohydrazide (**6i**)

Beige solid (151 mg, 0.309 mmol, 87%), mp. 278 °C. FT-IR (KBr): ν (cm^−1^) =3464, 3405, 3206, 3066, 2924, 2843, 1613, 1525, 1443, 1353, 1294, 1265, 1187, 1021, 936, 898, 872, 826, 758, 735, 683, and 521 cm^−1^. ^1^H-NMR (700 MHz, DMSO-*d*_6_), δ 12.35 (s, 1H), 11.60 (s, 1H), 8.39 (s, 1H), 7.73 (d, *J* = 8.4 Hz, 2H), 7.58 (d, *J* = 4.4 Hz, 1H), 7.52 (s, 1H), 7.49 (d, *J* = 7.6 Hz, 2H), 7.44 (d, *J* = 8.0 Hz, 1H), 7.25 (t, *J* = 8.6 Hz, 1H), 7.15 (s, 2H), 7.03 (s, 1H), 6.73 (d, *J* = 8.3 Hz, 2H), and 4.58 (d, *J* = 5.6 Hz, 2H) ppm. ^13^C-NMR (176 MHz, DMSO-*d*_6_), δ 163.49, 162.69 (^1^*J*_C-F_ = 245 Hz), 153.36, 151.95, 145.13, 143.67, 137.76 (^3^*J*_C-F_ = 7.8Hz), 134.79, 131.34 (^3^*J*_C-F_ = 8.5 Hz), 129.72, 123.67, 122.29, 121.55, 120.72, 118.87, 116.88 (^2^*J*_C-F_ = 21 Hz), 113.20 (^2^*J*_C-F_ = 22 Hz), 111.87, 111.70, and 41.75 ppm. Mass (ESI): *m*/*z* 388 [(^18^F)M+H]^+^; 389 [(^19^F)M+H]^+^.

#### 3.3.10. (*E*)-4-(((1*H*-benzo[d]imidazol-2-yl)methyl)amino)-*N*′-(4-fluorobenzylidene)benzohydrazide (**6j**)

White solid (70 mg, 0.180 mmol, 53%), mp. 291 °C. FT-IR (KBr): ν (cm^−1^) =3392, 2956, 2898, 2790, 2693, 1649,1604, 1502, 1438, 1298, 1187, 1145, 832, 751, 696, and 514 cm^−1^. ^1^H-NMR (700 MHz, DMSO-*d*_6_), δ 12.34 (s, 1H), 11.49 (s, 1H), 8.38 (s, 1H), 7.75 (s, 2H), 7.71 (d, *J* = 8.6 Hz, 2H), 7.55 (s, 1H), 7.46 (s, 1H), 7.28 (t, *J* = 8.7 Hz, 2H), 7.15 (s, 2H), 7.01 (t, *J* = 6.0 Hz, 1H), 6.73 (d, *J* = 8.6 Hz, 2H), and 4.57 (d, *J* = 5.8 Hz, 2H) ppm. ^13^C-NMR (176 MHz, DMSO-*d*_6_), δ ^13^C NMR (176 MHz, DMSO), δ 163.37, 163.35 (^1^*J*_C-F_ = 247 Hz), 153.38, 151.87, 145.44, 131.75, 129.62, 129.42 (^1^*J*_C-F_ = 8.5 Hz), 122.24, 121.58, 120.89, 118.87, 116.31 (^2^*J*_C-F_ = 22 Hz), 111.87, 111.68, and 41.76 ppm. Mass (ESI): *m*/*z* 388 [(^18^F)M+H]^+^; 389 [(^19^F)M+H]^+^.

#### 3.3.11. (*E*)-4-(((1*H*-benzo[d]imidazol-2-yl)methyl)amino)-*N*′-(2,4-dichlorobenzylidene)benzohydrazide (**6k**)

White solid (134 mg, 0.305 mmol, 86%), mp. 294 °C. FT-IR (KBr): ν (cm^−1^) = 3385, 3096, 2956, 2790, 1656, 1604, 1522l, 1441, 1330, 1298, 1258, 1184, 1044, 970, 822, 739, 692, 637, and 514 cm^−1^. ^1^H-NMR (700 MHz, DMSO-*d*_6_), δ ^1^H NMR (700 MHz, DMSO), δ 12.35 (s, 1H), 11.78 (s, 1H), 8.75 (s, 1H), 7.99 (s, 1H), 7.74 (d, *J* = 8.7 Hz, 2H), 7.71 (s, 1H), 7.57 (s, 1H), 7.51 (d, *J* = 8.8 Hz, 1H), 7.45 (s, 1H), 7.15 (s, 2H), 7.06 (t, *J* = 5.8 Hz, 1H), 6.74 (d, *J* = 8.7 Hz, 2H), and 4.59 (d, *J* = 5.7 Hz, 2H) ppm. ^13^C-NMR (176 MHz, DMSO-*d*_6_), δ 163.47, 153.42, 152.17, 143.77, 141.38, 135.20, 134.88, 134.11, 131.62, 129.89, 128.55, 128.46, 122.36, 121.68, 120.58, 118.99, 111.99, 111.80, and 41.83 ppm. Mass (ESI): *m*/*z* 438 [^35^(Cl)M+H]^+^, 440 [^37^(Cl)M+H]^+^.

#### 3.3.12. (*E*)-4-(((1*H*-benzo[d]imidazol-2-yl)methyl)amino)-*N*′-(2,5-difluorobenzylidene)benzohydrazide (**6l**)

Cream solid (110 mg, 0.271 mmol, 84%), mp. 160 °C. FT-IR (KBr): ν (cm^−1^) =3444, 3206, 3139, 2963, 2826, 1642, 1606, 1519, 1486, 1428, 1353, 1298, 1184, 1090, 1060, 1014, 940, 898, 836, 813, 739, and 481 cm^−1^. ^1^H-NMR (700 MHz, DMSO-*d*_6_) δ ^1^H NMR (700 MHz, DMSO) δ 12.36 (s, 1H), 11.72 (s, 1H), 8.59 (s, 1H), 7.73 (d, *J* = 8.5 Hz, 2H), 7.63–7.59 (m, 1H), 7.51 (dt, *J* = 9.8, 4.8 Hz, 2H), 7.39–7.36 (m, 1H), 7.32 (dt, *J* = 8.8, 4.3 Hz, 1H), 7.15 (dt, *J* = 7.5, 3.7 Hz, 2H), 7.06 (t, *J* = 5.7 Hz, 1H), 6.74 (d, *J* = 8.4 Hz, 2H), and 4.58 (d, *J* = 5.7 Hz, 2H) ppm. ^13^C-NMR (176 MHz, DMSO-*d*_6_), δ 164.49, 158.00 (dd, ^1^*J*_C-F_ = 277, 243 Hz), 157 (ddd, ^1^*J*_C-F_ = 245, 50, 2.4Hz), 153.32, 152.07, 138.07, 129.77, 124.35 (dd, ^1^*J*_C-F_ = 12.1, 7.8 Hz), 121.94, 121.75 (dd, ^1^*J*_C-F_ = 24, 9 Hz), 120.45, 119.34 (dd, ^1^*J*_C-F_ = 26, 8.4 Hz), 118.53 (dd, ^1^*J*_C-F_ = 24.6, 9 Hz), 118.34 (dd, ^1^*J*_C-F_ = 24, 8.4 Hz), 118.20, 118.05, 112.04, 112.03, 111.90, 111.88, and 41.72 ppm. Mass (ESI): *m*/*z* 406 [(^18^F)M+H]^+^; 407 [(^19^F)M+H]^+^.

### 3.4. Biological Evaluation

#### 3.4.1. In Vitro Cytotoxicity Assay

The cytotoxic potential of compounds **6a-l** was evaluated against a panel of cancer cell lines, including HCT-116, HepG2, and MCF-7, as well as a normal cell line, WI-38, using the reported methodology [44]. The cell lines utilized in this study were obtained from the American Type Culture Collection (ATCC) through the Holding Company for Biological Products and Vaccines (VACSERA) in Cairo, Egypt. These included: 1. A human colon cancer cell line (HCT-116); 2. Hepatocellular carcinoma (HepG2)—a liver cancer cell line; 3. Mammary gland breast cancer (MCF-7); and 4. A human lung fibroblast (WI-38)—a normal cell line.

#### 3.4.2. In Vitro Enzyme Inhibitory Assays

The enzyme inhibitory activities of compounds **6c** and **6h-j** against EGFR, HER2, VEGFR2, CDK2, AURKC, HDAC1, and mTOR enzymes were assessed using recently reported methods [44].

#### 3.4.3. Cell Cycle Analysis

Cell cycle distribution was evaluated using ab139418 Propidium Iodide (PI) flow cytometry kit/BD following a previously reported method [44,45] for the most potent compound **6i**.

#### 3.4.4. Annexin-V/Propidium Iodide (PI) Double Staining Assay

The apoptosis effect of compound **6i** was studied using a previously reported method [44,45]. 

#### 3.4.5. Determination of Apoptotic Protein Levels

Apoptotic protein levels for compound **6i** were determined using an ELISA technique for caspase-3, BAX, and Bcl-2, applying a previously reported method [44,46,47].

### 3.5. In Silico Molecular Docking Studies

Molecular docking analysis of compounds **6h** and **6i** were conducted using the active sites of EGFR (PDB: 4HJO), Her2 (PDB: 7PCD), and AURKC (PDB: 6GR8) kinase enzymes. For docking studies, MOE 2020 software programs were employed.

## 4. Conclusions

The findings of this study highlight the significant potential of the novel class of halogenated compounds with the structural motif “(*E*)-4-(((1*H*-Benzo[*d*]imidazol-2-yl)methyl)amino)-*N*′-(halogenated)benzohydrazide (**6a-l**)” as promising candidates for the development of highly potent and effective targeted kinase inhibitors (TKIs). The in vitro evaluation of this compound series demonstrated excellent cytotoxic effects against a panel of diverse cancer cell lines, with select analogs, such as **6c** and **6h-j**, exhibiting remarkably low IC_50_ values in the range of 7.82 to 21.48 μM. Of particular note, compounds **6h** and **6i** emerged as the most potent inhibitors, displaying significant inhibitory activity against critical oncogenic kinases, including EGFR, HER2, CDK2, AURKC, and mTOR. The in-depth mechanistic investigation of lead compound **6i** uncovered its remarkable capability to induce cell cycle arrest and programmed cell death (apoptosis) within HepG2 liver cancer cells. This was accompanied by a notable upregulation of pro-apoptotic proteins caspase-3 and Bax, coupled with a downregulation of the anti-apoptotic protein Bcl-2. Furthermore, computational molecular docking analyses suggested favorable binding interactions between compounds **6h** and **6i** and the relevant target enzymes. These findings collectively underscore the immense potential of this class of halogenated-1*H*-benzo[*d*]imidazol-2-yl)methyl)amino)-*N*′-benzohydrazide compounds as a promising avenue for the development of next-generation multi-targeted kinase inhibitors with enhanced potency and therapeutic efficacy. The ability of these compounds to modulate multiple oncogenic signaling pathways simultaneously presents a compelling opportunity to address the inherent complexity and adaptability of cancer cells, potentially overcoming the limitations associated with single-target kinase inhibitors. Moving forward, further optimization of the lead compounds, including structural modifications, in-depth mechanistic studies, and rigorous in vivo evaluations, will be crucial to fully elucidate the therapeutic potential of this compound class. Successful translation of these findings into clinical settings could pave the way for the development of innovative treatment strategies, ultimately benefiting patients with a wide range of cancers and other diseases characterized by deregulated kinase signaling.

## Figures and Tables

**Table 1:** In vitro cytotoxicity of compounds **6a-l** against selected cancer cell lines.

Compound		In Vitro Cytotoxicity IC_50_ (µM)						
No.	R	HCT-116	SI	HepG2	SI	MCF-7	SI	WI-38
**6a**	H	24.62 ± 1.9	2.95	31.76 ± 2.2	2.29	26.31 ± 1.9	2.76	72.60 ± 4.0
**6b**	2-Br	42.90 ± 2.5	1.96	26.16 ± 1.9	3.22	31.82 ± 2.1	2.64	84.15 ± 4.3
**6c**	3-Br	10.21 ± 0.8	4.72	8.90 ± 0.6	5.41	7.82 ± 0.6	6.16	48.17 ± 2.7
**6d**	4-Br	30.26 ± 2.1	1.79	17.78 ± 1.3	3.05	25.18 ± 1.8	2.16	54.30 ± 3.1
**6e**	2-Cl	46.67 ± 2.7	1.33	28.80 ± 1.7	2.15	36.16 ± 2.3	1.71	61.86 ± 3.5
**6f**	3-Cl	63.72 ± 3.5	1.57	34.79 ± 2.1	2.87	45.61 ± 2.5	2.19	>100
**6g**	4-Cl	35.29 ± 2.3	2.02	24.90 ± 1.7	2.86	22.51 ± 1.6	3.16	71.19 ± 3.5
**6h**	2-F	21.48 ± 1.6	4.33	12.94 ± 1.0	7.19	16.31 ± 1.2	5.70	92.98 ± 4.6
**6i**	3-F	13.44 ± 1.2	4.20	9.39 ± 0.8	6.01	11.64 ± 0.9	4.85	56.46 ± 3.0
**6j**	4-F	18.72 ± 1.4	2.08	14.02 ± 1.2	2.78	8.31 ± 0.7	4.70	39.03 ± 2.3
**6k**	2,4-di-Cl	67.83 ± 3.8	1.47	44.78 ± 2.4	2.23	48.64 ± 2.7	2.06	>100
**6l**	2,5-di-F	39.84 ± 2.2	1.95	20.02 ± 1.5	3.88	29.81 ± 2.0	2.60	77.61 ± 4.1
Sorafenib		5.47 ± 0.3	1.95	9.18 ± 0.6	1.16	7.26 ± 0.3	1.47	10.65 ± 0.8
Doxorubicin		5.23 ± 0.3	1.28	4.50 ± 0.2	1.49	4.17 ± 0.2	1.61	6.72 ± 0.5
Sunitinib		17.91 ± 1.3	3.11	8.38 ± 0.5	6.64	24.06 ± 2.0	2.31	55.63 ± 3.3

IC_50_ values are the mean ± SD of triplicate measurements; SI = Selectivity Index = IC_50_ of normal cell line (WI-38)/IC_50_ of cancer cell line [41].

**Table 2:** In vitro protein kinase inhibition of compounds **6c** and **6h-j** against EGFR, Her2, VEGFR2, CDK2, AURKC, HDAC1, and mTOR.

Compound	In Vitro Protein Kinase Inhibition IC_50_ (nM)						
	EGFR	Her2	VEGFR2	CDK2	AURKC	HDAC1	mTOR
**6c**	125.2 ± 0.041	55.6 ± 0.023	604.5 ± 0.022	938 ± 0.039	94.4 ± 0.036	2263 ± 0.077	1461 ± 0.05
**6h**	73.2 ± 0.004	23.2 ± 0.001	194.5 ± 0.007	284 ± 0.012	11 ± 0.004	151.1 ± 0.005	413 ± 0.014
**6i**	30.1 ± 0.03	28.3 ± 0.001	172.2 ± 0.006	364 ± 0.011	74.5 ± 0.003	96.6 ± 0.003	152 ± 0.005
**6j**	166.4 ± 0.008	204.7 ± 0.009	307.2 ± 0.011	1448 ± 0.062	589.4 ± 0.022	473.3 ± 0.016	1305 ± 0.044
**Erlotinib**	61.1 ± 0.002	-	-	-	-	-	-
**Lapatenib**	-	17.4 ± 0.001	-	-	-	-	-
**Sorafenib**	-	-	45.4 ± 0.002	-	-	-	-
**Roscovetine**	-	-	-	756 ± 0.032	-	-	-
**TSA**	-	-	-	-	30.4 ± 0.001	37.4 ± 0.001	-
**Rapamycin**	-	-	-	-	-	-	208 ± 0.007

IC_50_ values are the mean ± SD of triplicate measurements.

**Table 3:** Results of cell cycle analysis of HepG2 cells treated with compound **6i**.

Compound/Cell Line	DNA Content (%)			Cell Cycle Distribution Index (CDI)
	%G0-G1	%S	%G2/M	
Cont. HepG2	52.39	34.77	12.84	0.91
Compound **6i**/HepG2	72.13	25.19	2.68	0.39

**Table 4:** Apoptotic cell distributions of HepG2 cells treated with compound **6i**.

Sample	Apoptosis			Necrosis
	Alive Cell (%)	Early	Late	
Cont. HepG2	97.58	0.61	0.21	1.6
Compound **6i**/HepG2	63.93	22.07	9.98	4.02

**Table 5:** Expression changes in apoptotic protein following treatment with compound **6i**.

Kinase Protein	Protein Expression (Pg/mL) (Folds)		
	Caspase-3	Bax	Bcl-2
Control HepG2	99.904 ± 3.88 (1)	71.075 ± 2.762 (1)	15.668 ± 0.53 (1)
**6i**/HepG2	388.497 ± 15.09 (3.9)	513.731 ± 19.96 (7.22)	2.073 ± 0.07 (0.132)
Saurosporine/HepG2	541.162 ± 21.02 (5.4)	386.743 ± 15.03 (5.44)	3.336 ± 0.11 (0.212)

Protein expression values are the mean ± SD of triplicate measurements.

## Data Availability

Data are given in the Supplementary Materials and are available upon request.

## Supplementary Materials

The following supporting information can be downloaded at https://www.mdpi.com/article/10.3390/ph17070839/s1: ^1^H-NMR, ^13^CNMR, and Mass spectra.
