# Supplementary material for: Discovery of 1H-benzo[d]imidazole-(halogenated)Benzylidenebenzohydrazide Hybrids as Potential Multi-Kinase Inhibitors
Source: Pharmaceuticals (Basel). 2024 Jun 26;17(7):839. doi: 10.3390/ph17070839 (PMC11279404; doi:10.3390/ph17070839)

Discovery of 1*H*-benzo[*d*]imidazole-(halogenated)Benzylidenebenzohydrazide Hybrids as Potential Multi Kinase Inhibitors

Tebyan O. Mirgany, Hanadi H. Asiri, A. F. M. Motiur Rahman* and Mohammed M. Alanazi*

Department of Pharmaceutical Chemistry, College of Pharmacy, King Saud University, Riyadh 11451, Saudi Arabia

Corresponding authors: mmalanazi@ksu.edu.sa (M. M. Alanazi); afmrahman@ksu.edu.sa (A. F. M. M. Rahman)

Content	Page no.	Content	Page no.
^{1}H-NMR spectra of compound **6a**	3	^{1}H-NMR spectra of compound **6g**	21
^{13}C-NMR spectra of compound **6a**	4	^{13}C-NMR spectra of compound **6g**	22
Mass spectra of compound **6a**	5	Mass spectra of compound **6g**	23
^{1}H-NMR spectra of compound **6b**	6	^{1}H-NMR spectra of compound **6h**	24
^{13}C-NMR spectra of compound **6b**	7	^{13}C-NMR spectra of compound **6h**	25
Mass spectra of compound **6b**	8	Mass spectra of compound **6h**	26
^{1}H-NMR spectra of compound **6c**	9	^{1}H-NMR spectra of compound **6i**	27
^{13}C-NMR spectra of compound **6c**	10	^{13}C-NMR spectra of compound **6i**	28
Mass spectra of compound **6c**	11	Mass spectra of compound **6i**	29
^{1}H-NMR spectra of compound **6d**	12	^{1}H-NMR spectra of compound **6j**	30
^{13}C-NMR spectra of compound **6d**	13	^{13}C-NMR spectra of compound **6j**	31
Mass spectra of compound **6d**	14	Mass spectra of compound **6j**	32
^{1}H-NMR spectra of compound **6e**	15	^{1}H-NMR spectra of compound **6k**	33
^{13}C-NMR spectra of compound **6e**	16	^{13}C-NMR spectra of compound **6k**	34
Mass spectra of compound **6e**	17	Mass spectra of compound **6k**	35
^{1}H-NMR spectra of compound **6f**	18	^{1}H-NMR spectra of compound **6l**	36
^{13}C-NMR spectra of compound **6f**	19	^{13}C-NMR spectra of compound **6l**	37
Mass spectra of compound **6f**	20	Mass spectra of compound **6l**	38

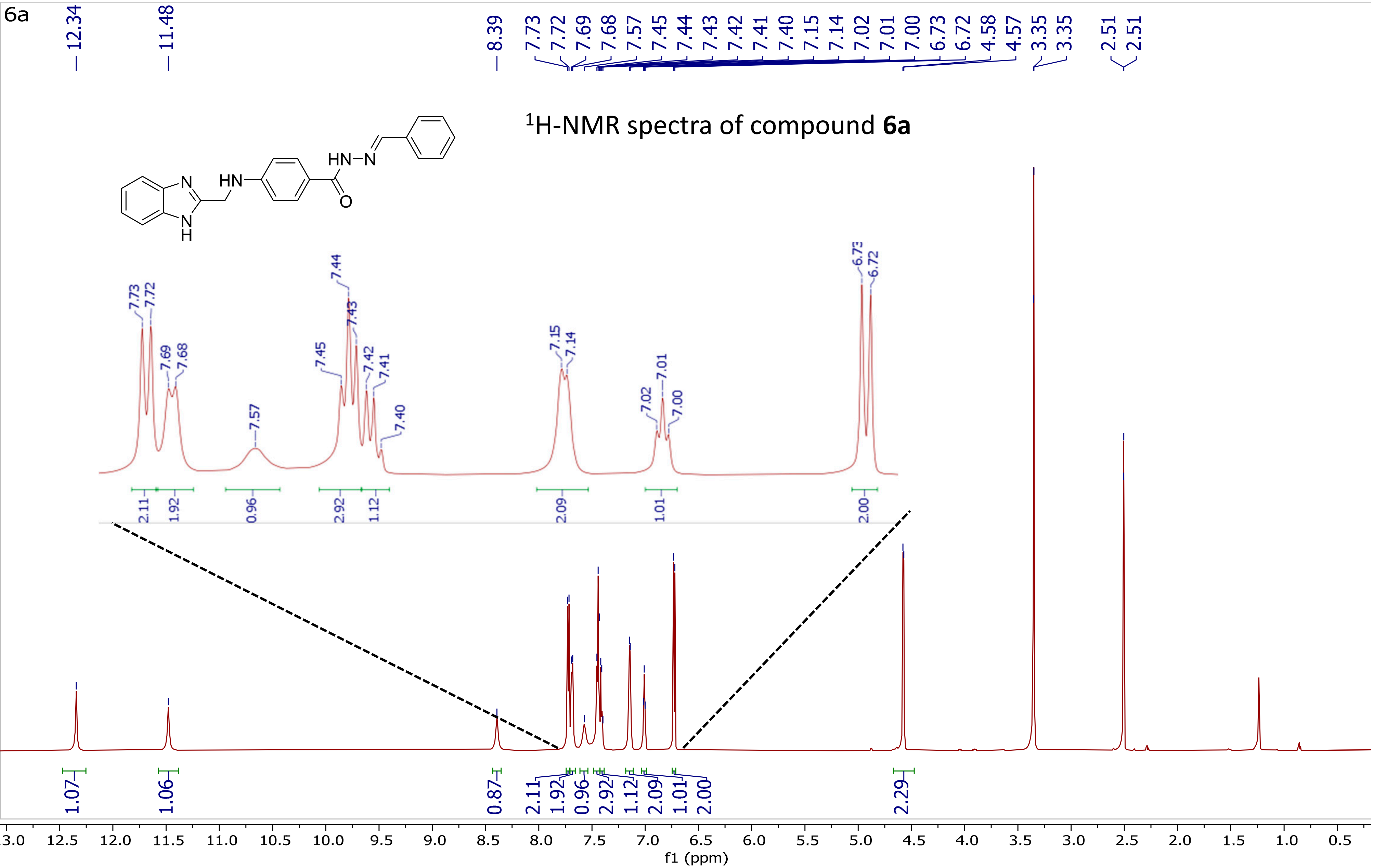
6a
12.34
11.48
8.39
7.73 7.72 7.69 7.68 7.57 7.45 7.44 7.43 7.42 7.41 7.40 7.15 7.14 7.02 7.01 7.00 6.73 6.72 4.58 4.57 3.35 3.35 2.51 2.51
¹H-NMR spectra of compound 6a
HN–N
N
HN
N
H
O
2.11 1.92 0.96 2.92 1.12 2.09 1.01 2.00
1.07 1.06 0.87 2.11 1.92 0.96 2.92 1.12 2.09 1.01 2.00 2.29
f1 (ppm)

6a

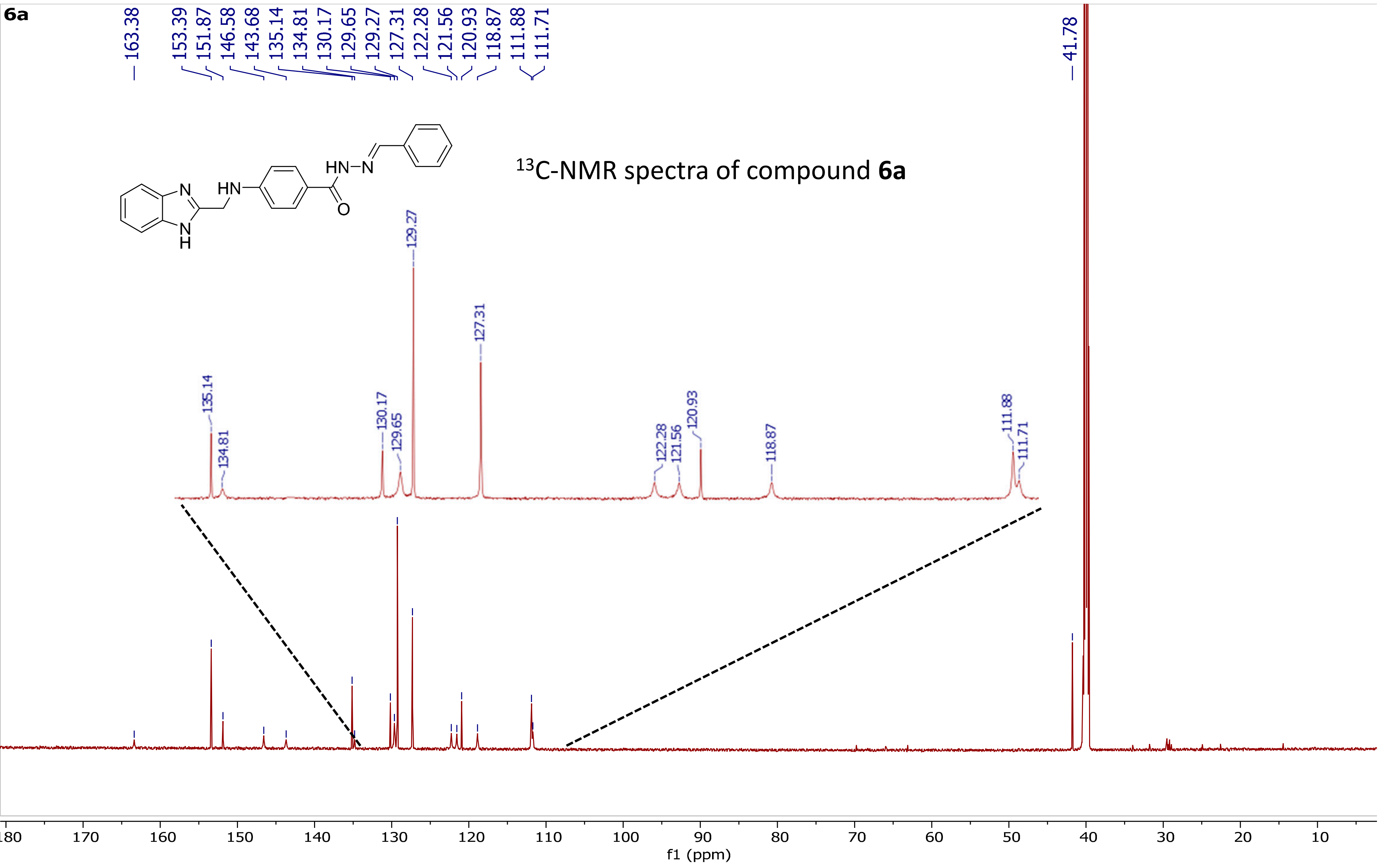
163.38
153.39
151.87
146.58
143.68
135.14
134.81
130.17
129.65
129.27
127.31
122.28
121.56
120.93
118.87
111.88
111.71
41.78
HN
N
HN
N
N
H
O
^{13}C-NMR spectra of compound **6a**
f1 (ppm)

Mass spectra of compound **6a**

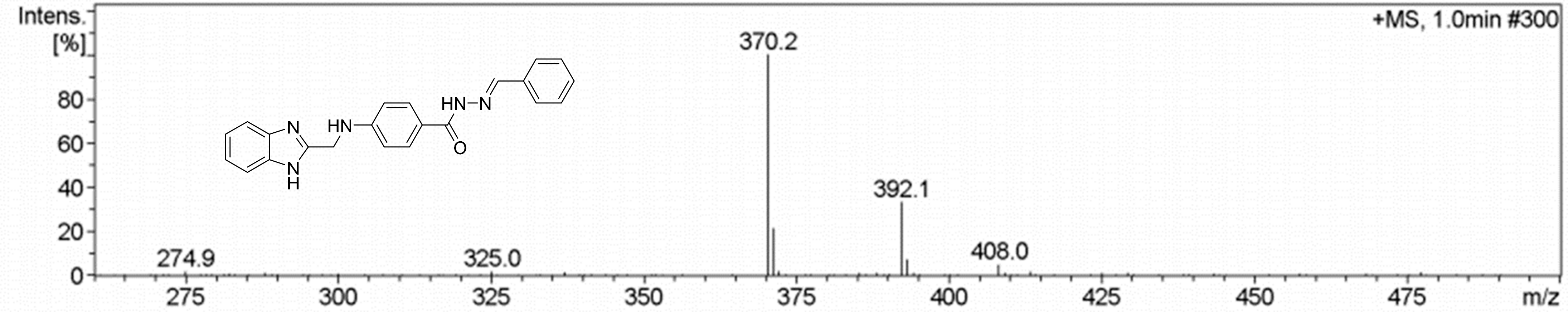

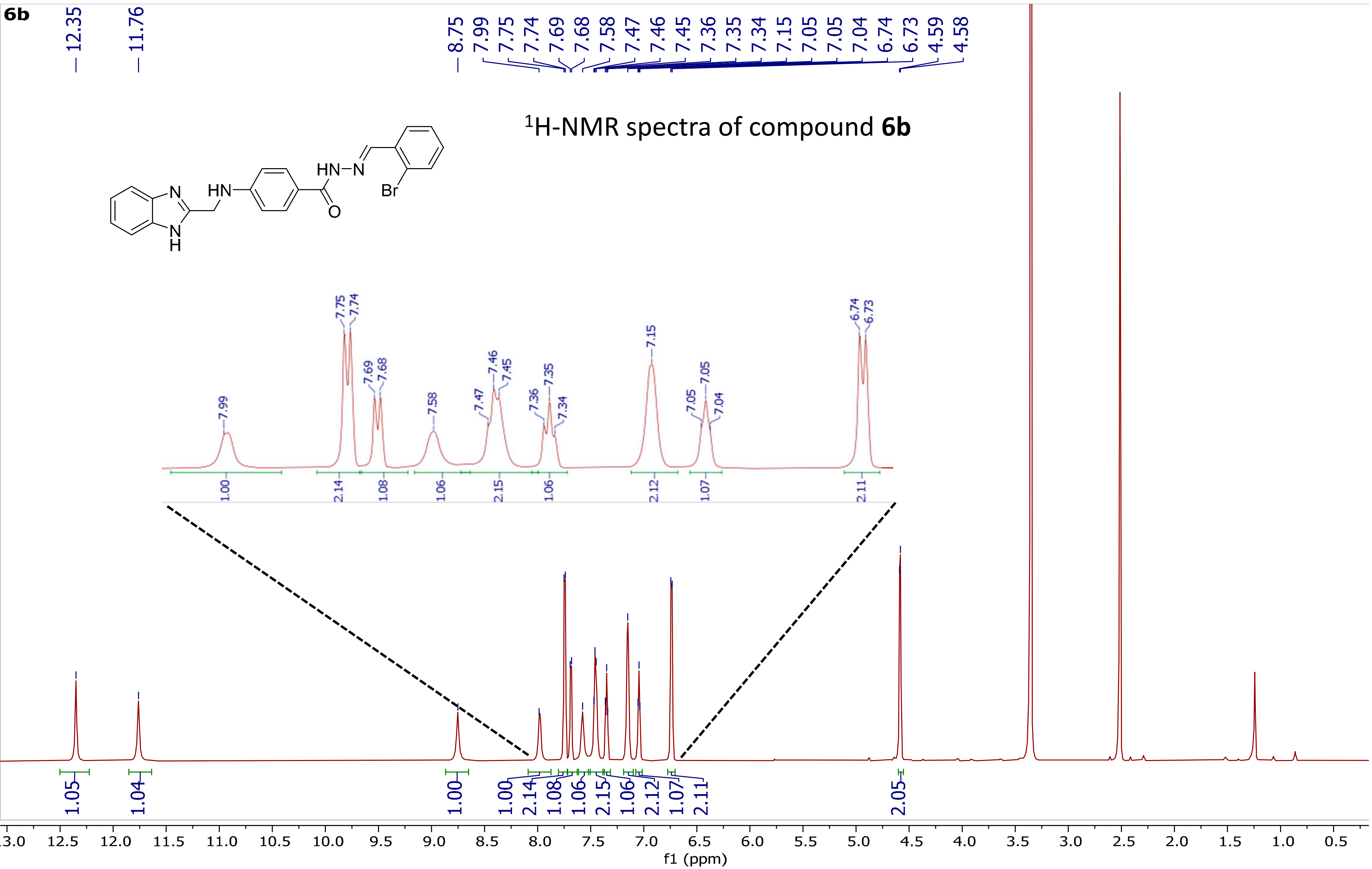
6b
1H-NMR spectra of compound 6b
HN
N
N
H
HN–N
O
Br
12.35
11.76
8.75
7.99
7.75
7.74
7.69
7.68
7.58
7.47
7.46
7.45
7.36
7.35
7.34
7.15
7.05
7.05
7.04
6.74
6.73
4.59
4.58
1.05
1.04
1.00
1.00
2.14
1.08
1.06
2.15
1.06
2.12
1.07
2.11
2.05
f1 (ppm)

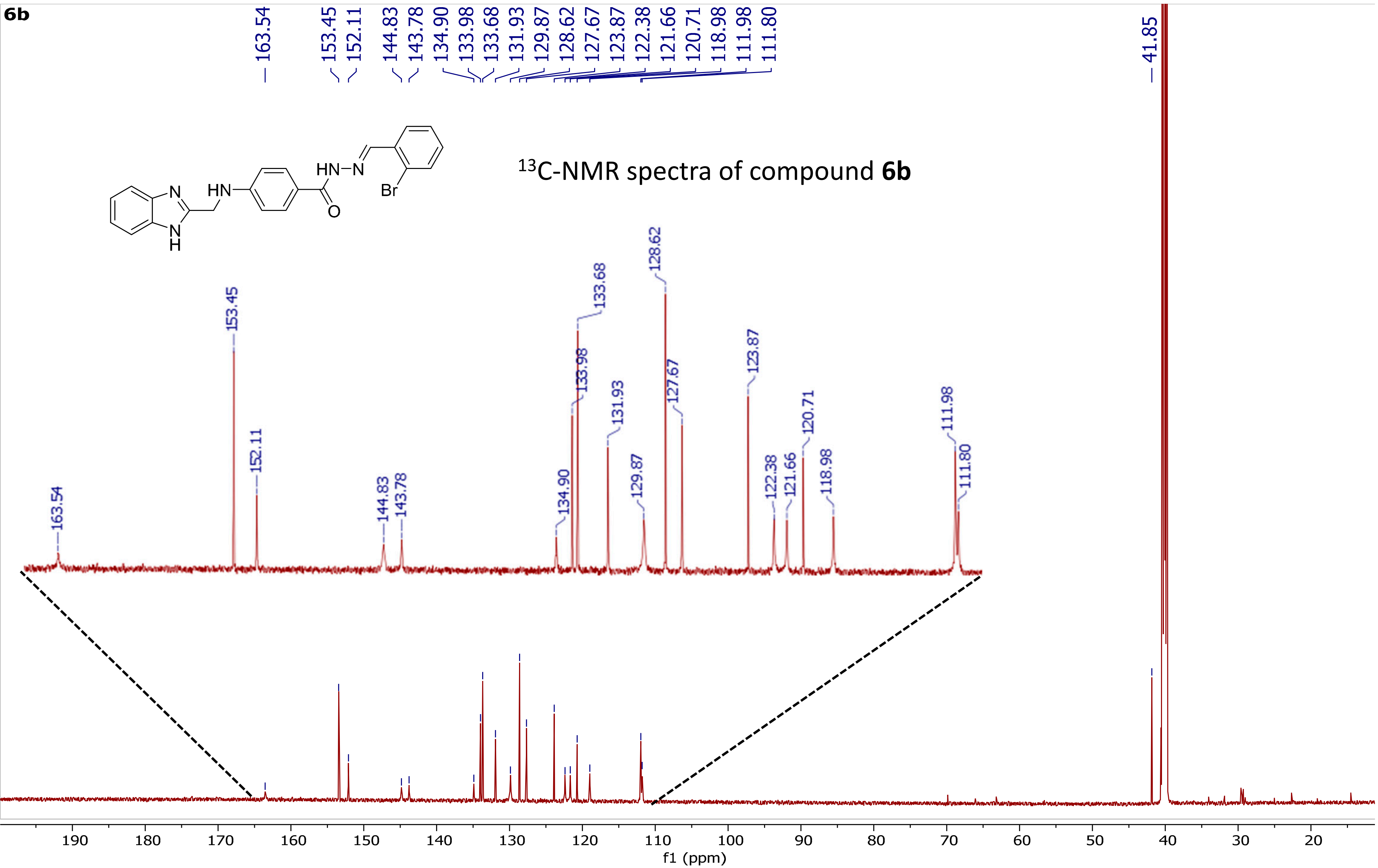
6b
163.54
153.45
152.11
144.83
143.78
134.90
133.98
133.68
131.93
129.87
128.62
127.67
123.87
122.38
121.66
120.71
118.98
111.98
111.80
41.85
HN
N
N
H
HN–N
O
Br
^{13}C-NMR spectra of compound **6b**
190
180
170
160
150
140
130
120
110
100
90
80
70
60
50
40
30
20
f1 (ppm)

Mass spectra of compound **6b**

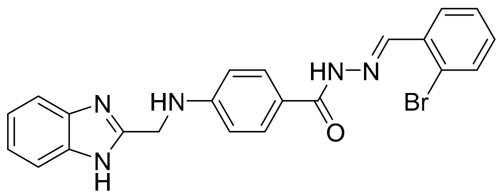

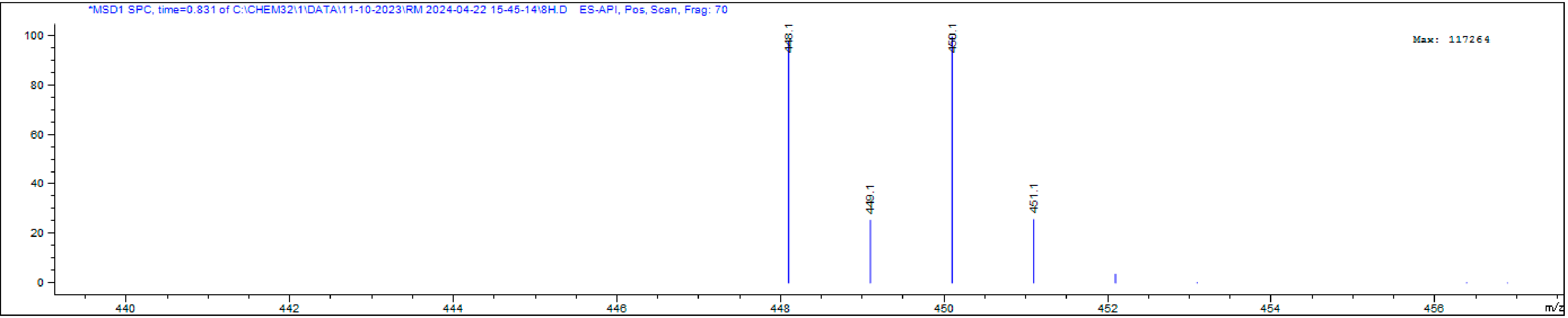

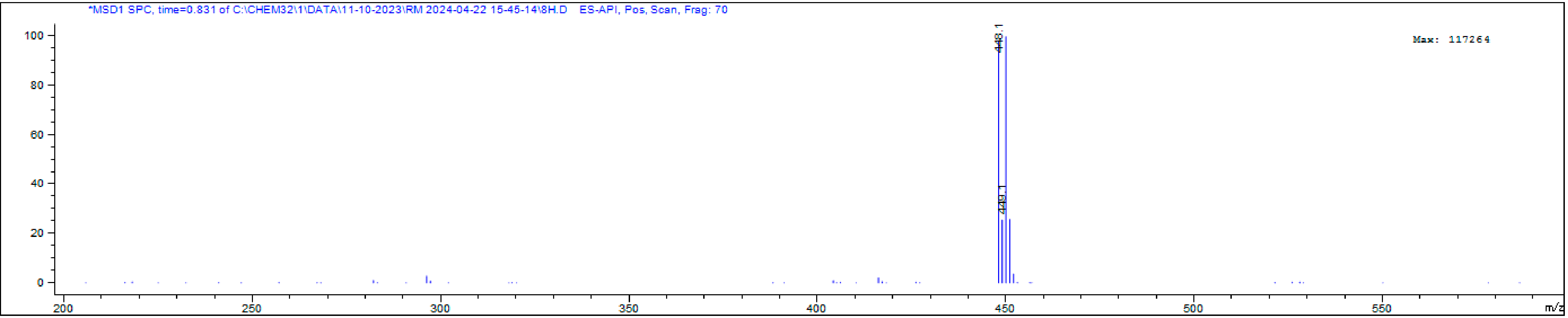

6c

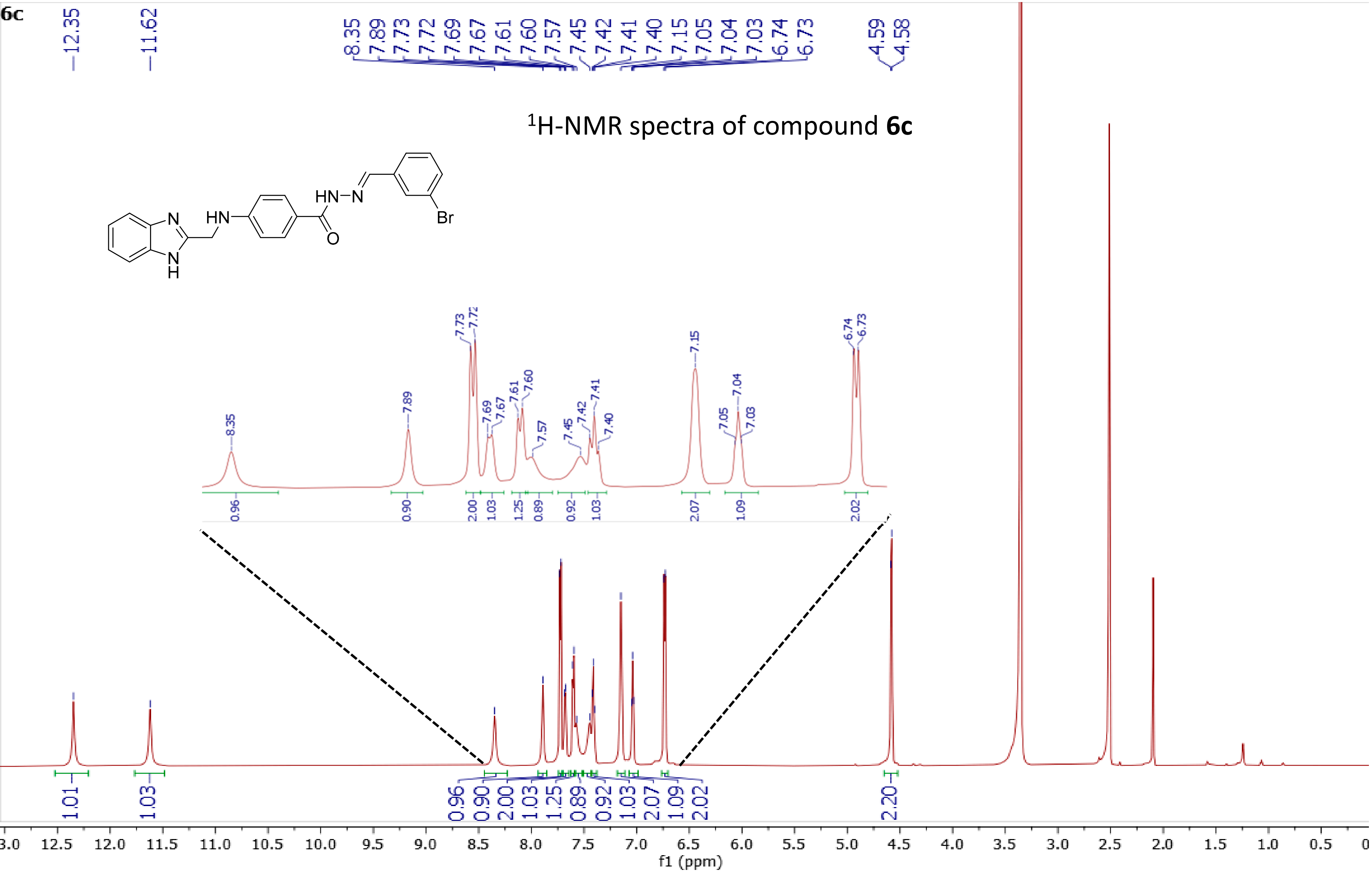

1H-NMR spectra of compound 6c
12.35
11.62
8.35
7.89
7.73
7.72
7.69
7.67
7.61
7.60
7.57
7.45
7.42
7.41
7.40
7.15
7.05
7.04
7.03
6.74
6.73
4.59
4.58
HN
N
N
H
HN–N
O
Br
1.01
1.03
0.96
0.90
2.00
1.03
1.25
0.89
0.92
1.03
2.07
1.09
2.02
2.20
f1 (ppm)

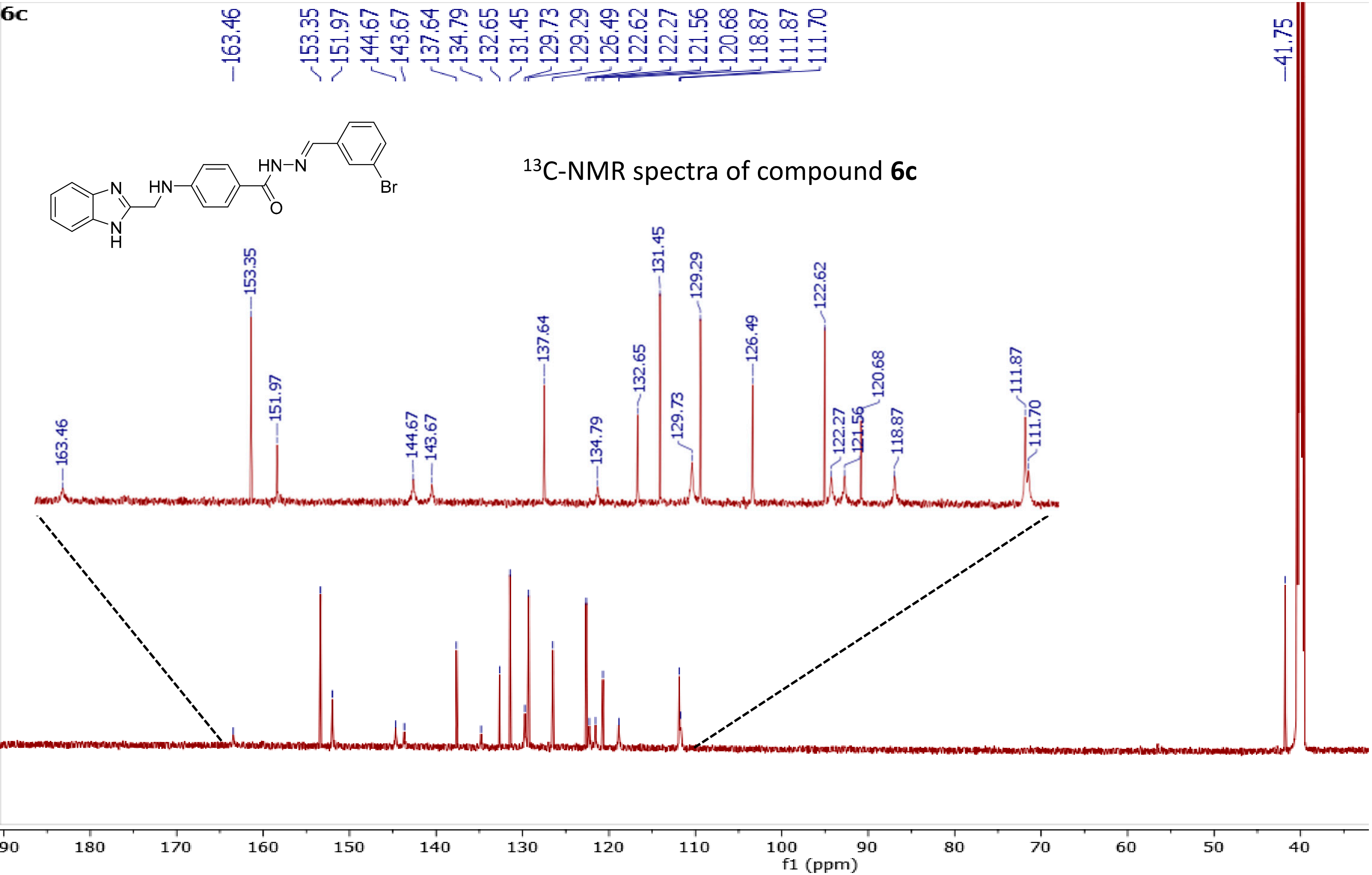

^{13}C-NMR spectra of compound **6c**

Mass spectra of compound **6c**

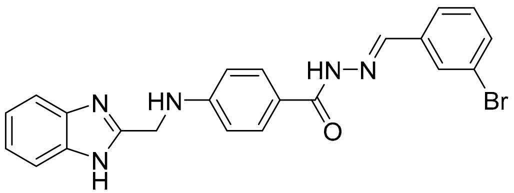

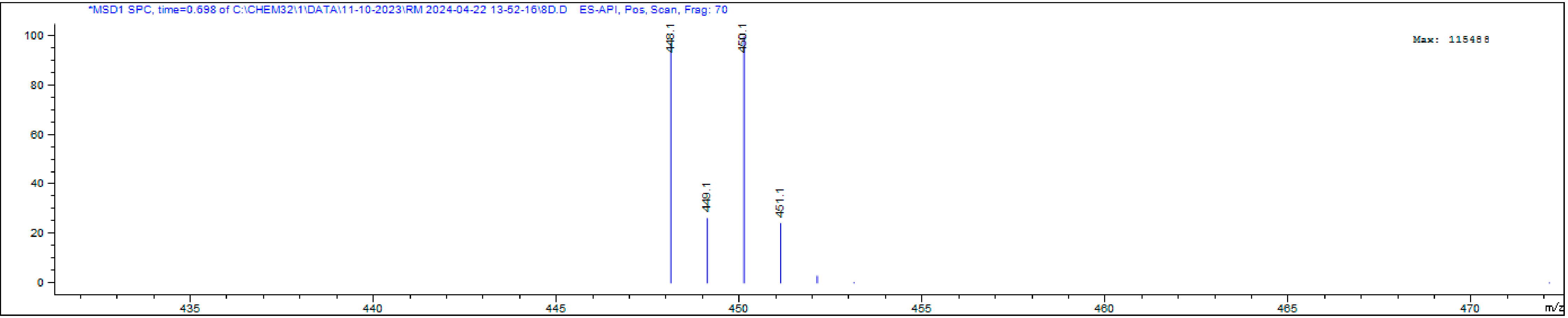

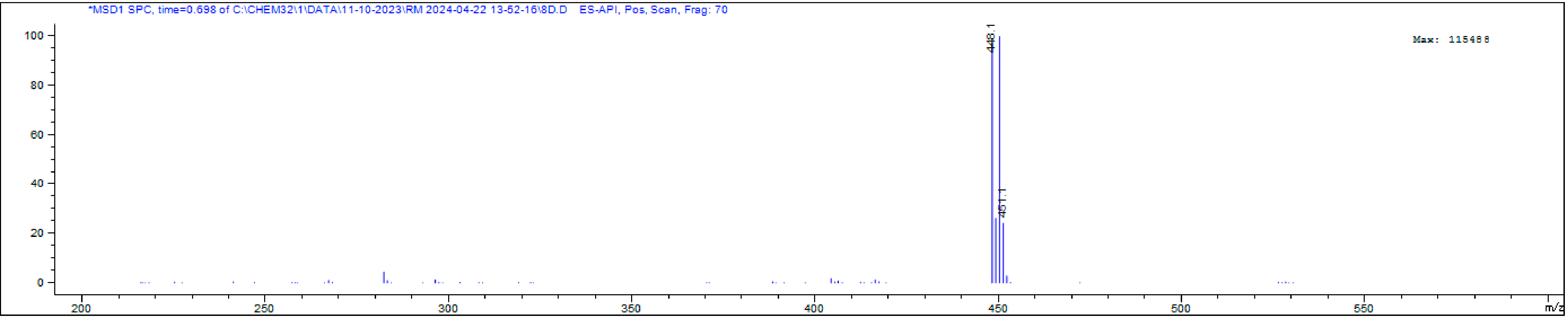

6d

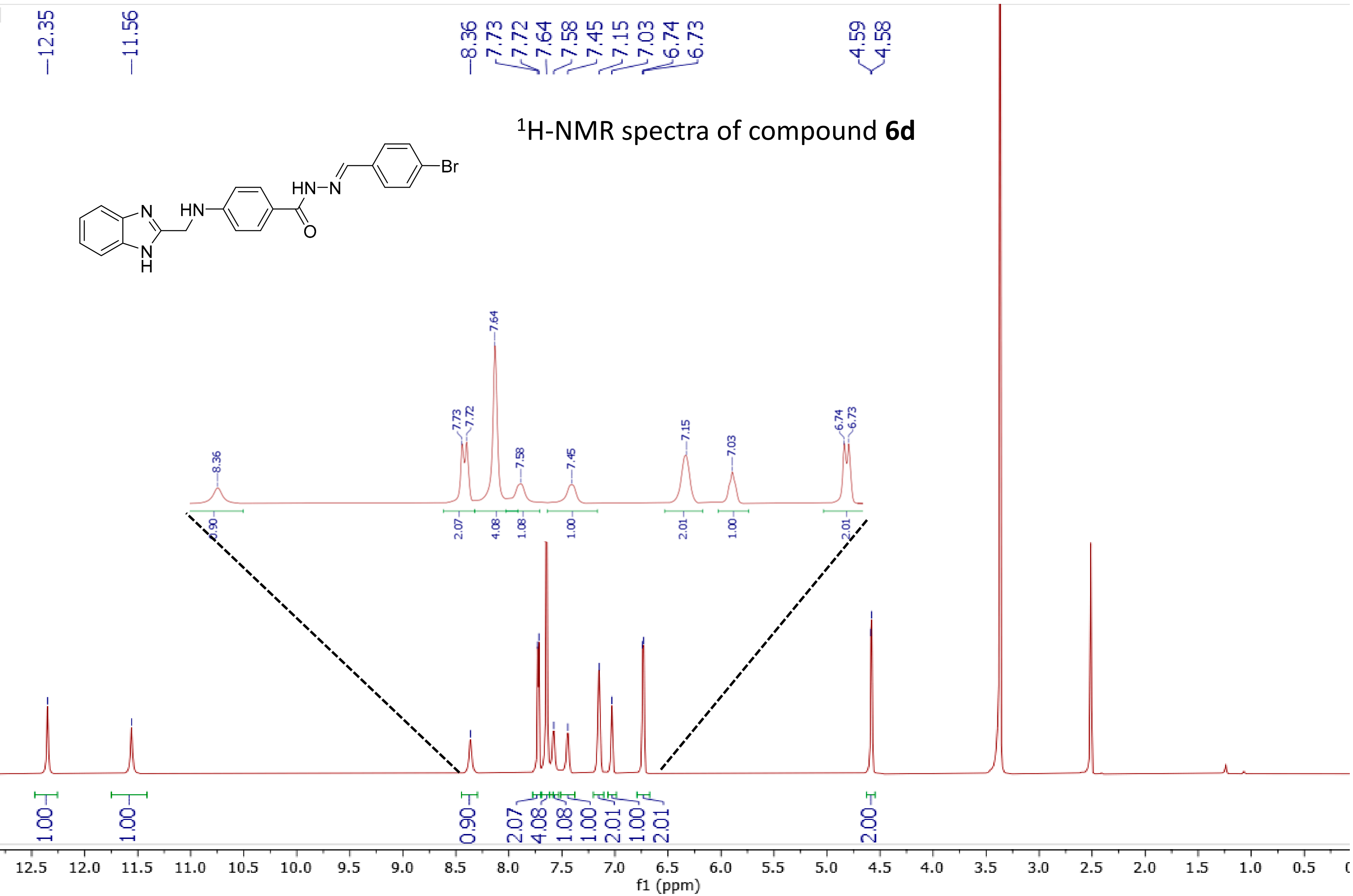
12.35
11.56
8.36
7.73
7.72
7.64
7.58
7.45
7.15
7.03
6.74
6.73
4.59
4.58
^{1}H-NMR spectra of compound **6d**
Br
HN–N
HN
N
N
H
O
0.90
2.07
4.08
1.08
1.00
2.01
1.00
2.01
1.00
1.00
0.90
2.07
4.08
1.08
1.00
2.01
1.00
2.01
2.00
f1 (ppm)

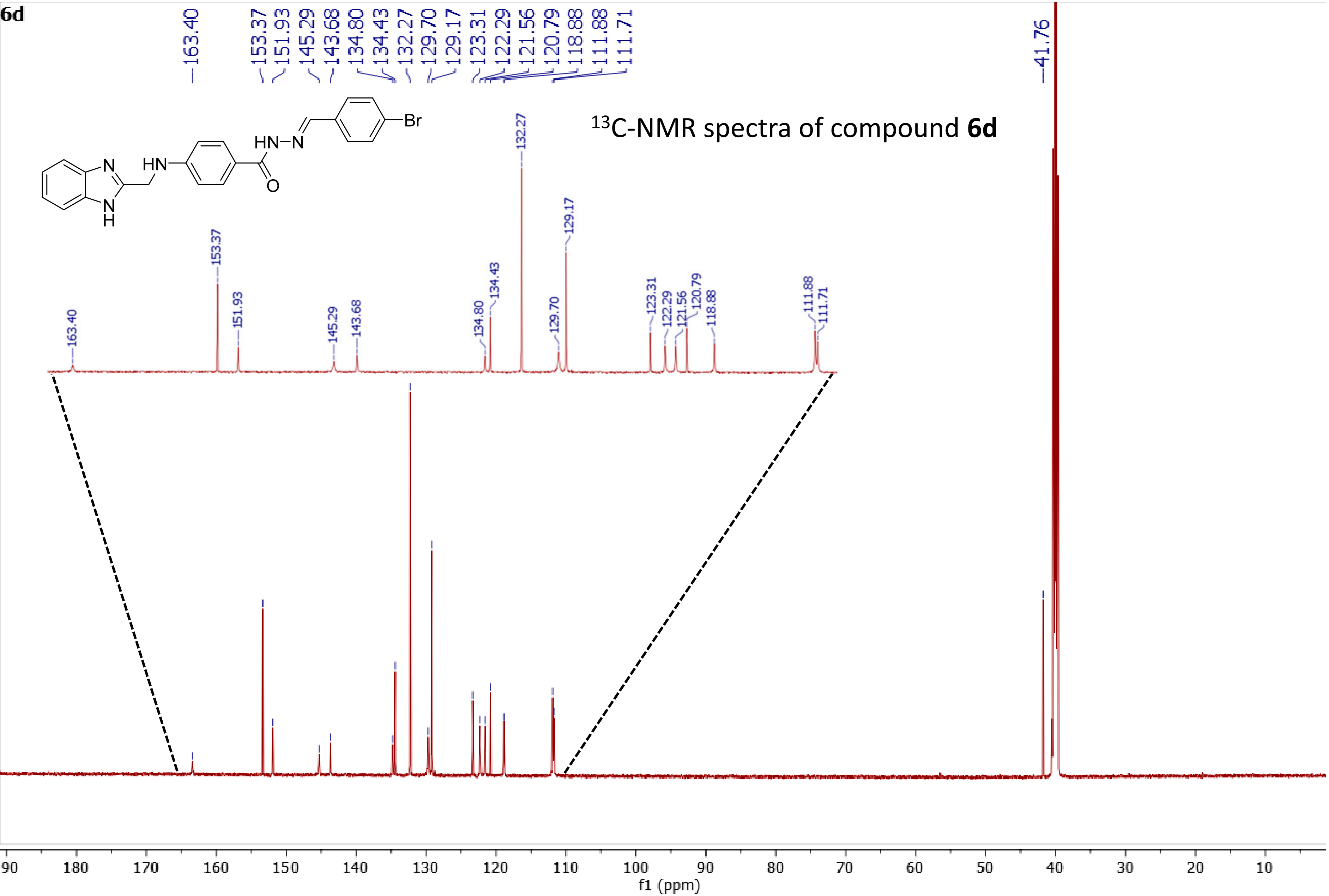

^{13}C-NMR spectra of compound **6d**

Mass spectra of compound **6d**

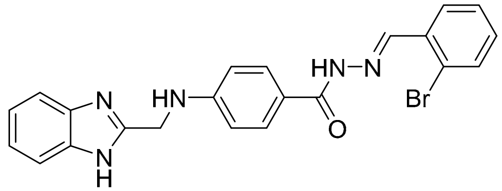

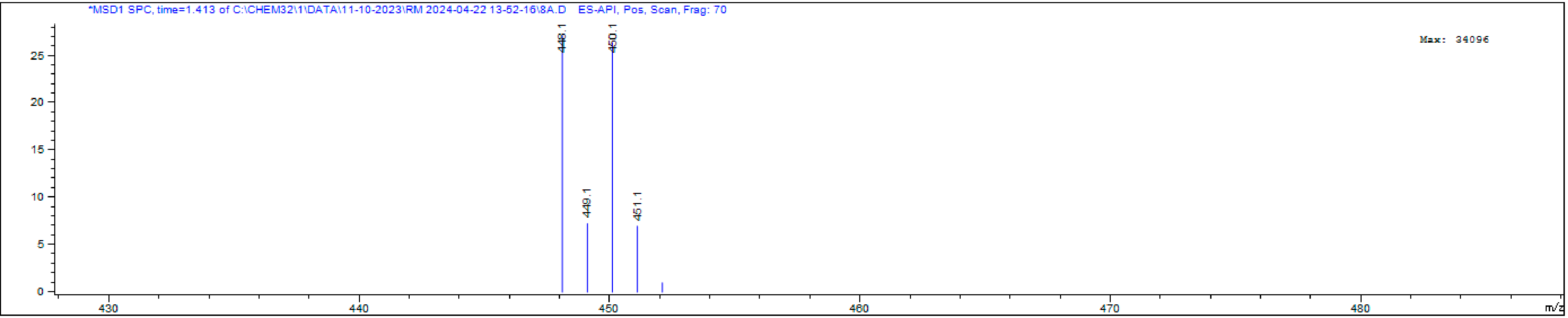

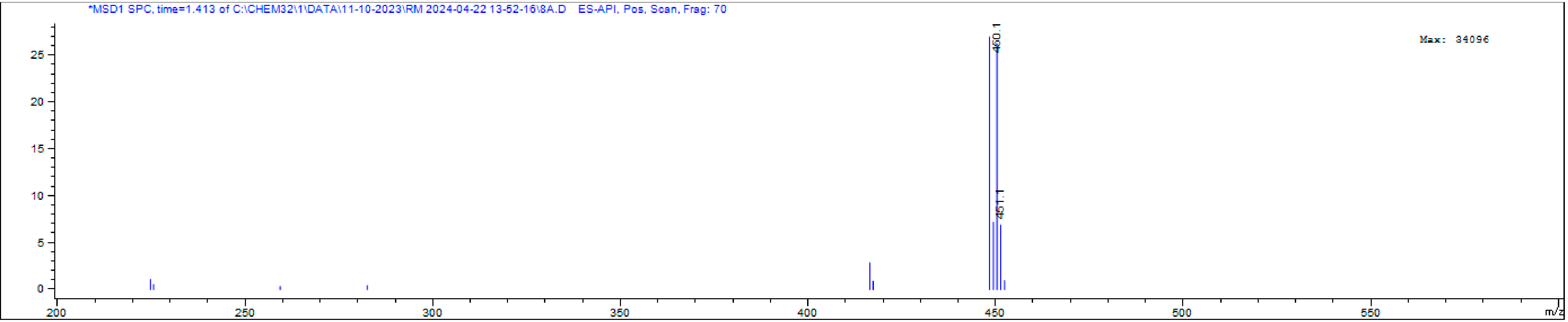

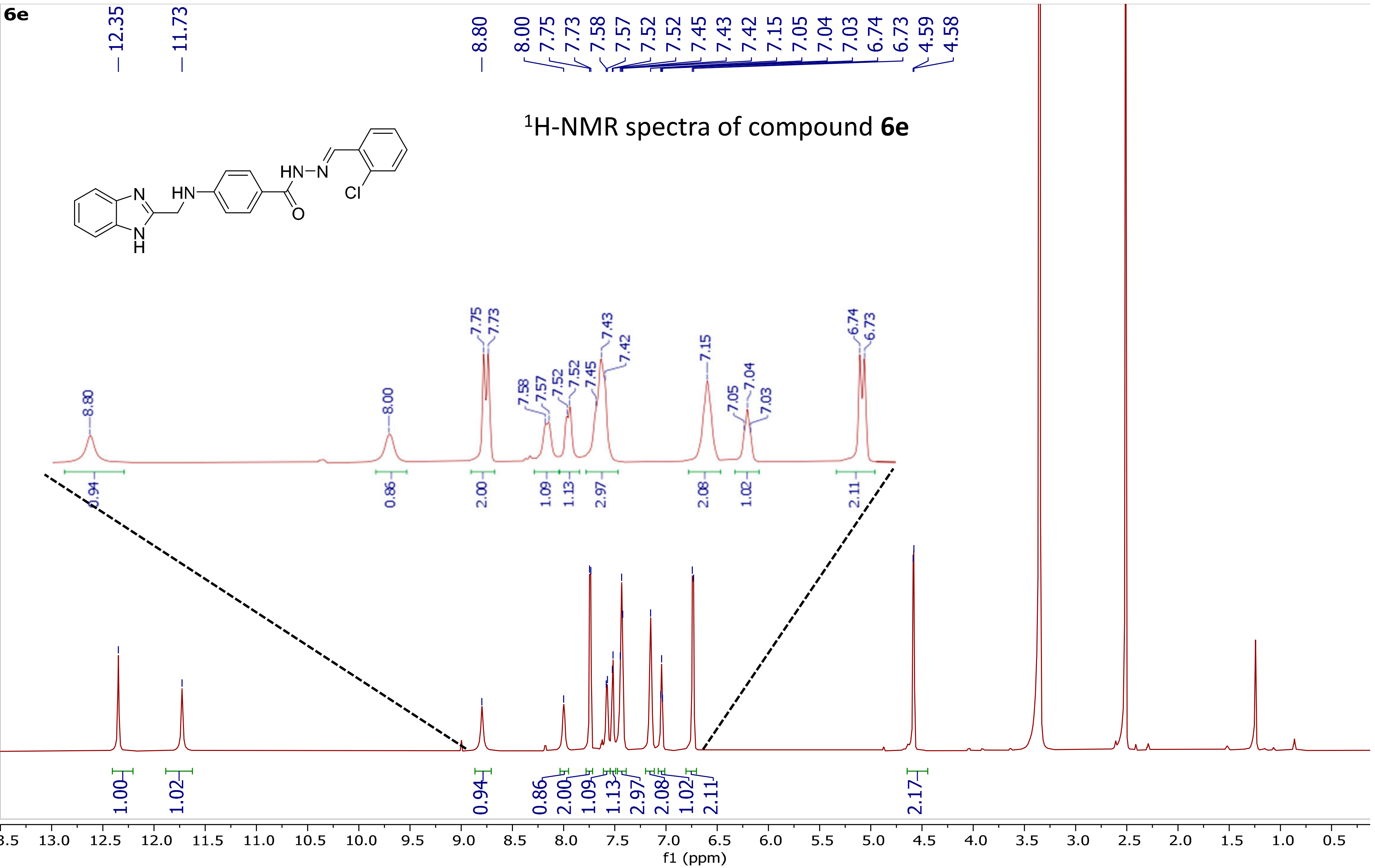
6e
1H-NMR spectra of compound 6e
f1 (ppm)

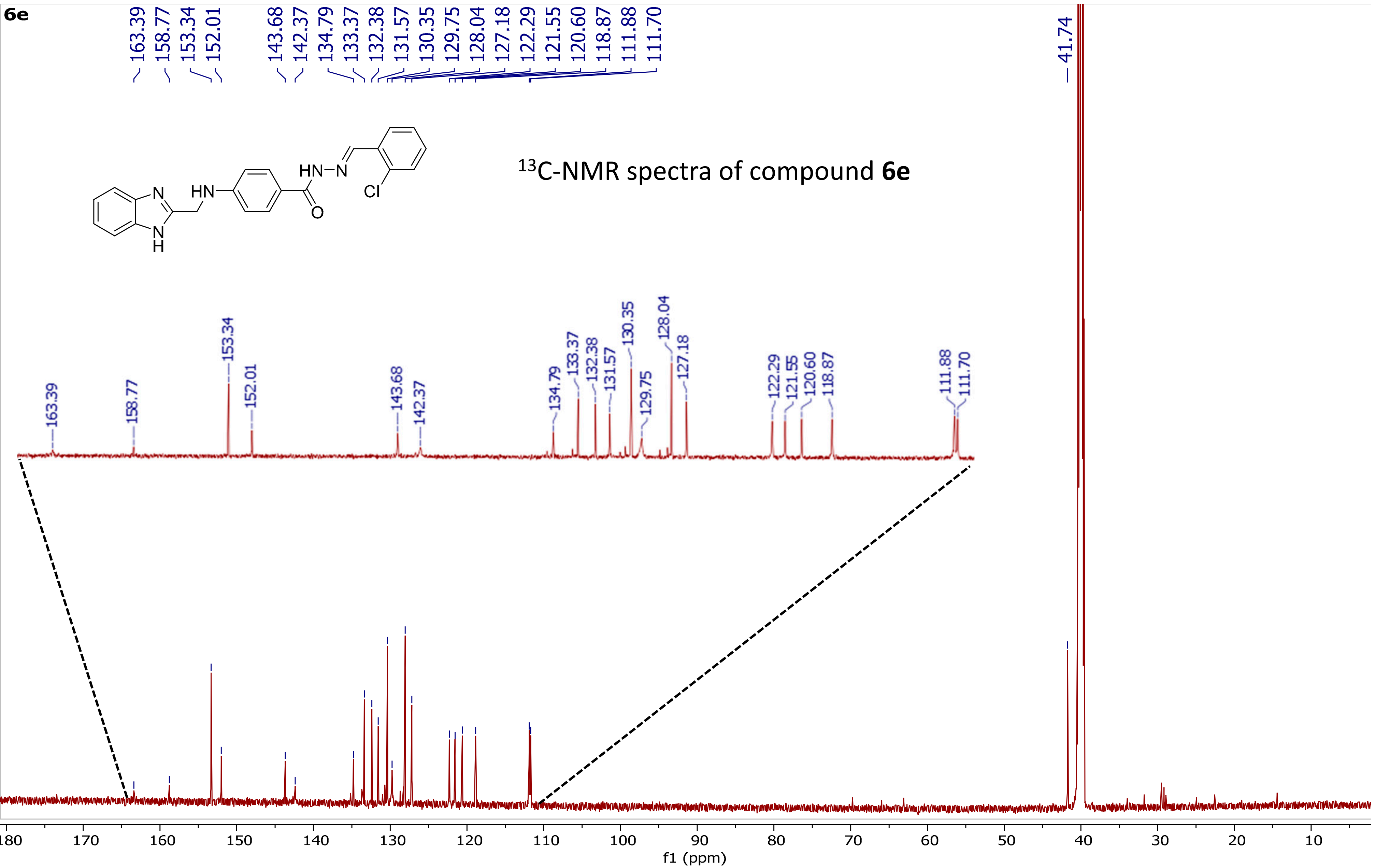
6e
163.39
158.77
153.34
152.01
143.68
142.37
134.79
133.37
132.38
131.57
130.35
129.75
128.04
127.18
122.29
121.55
120.60
118.87
111.88
111.70
41.74
^{13}C-NMR spectra of compound **6e**
HN
HN–N
N
N
H
O
Cl
f1 (ppm)

Mass spectra of compound **6e**

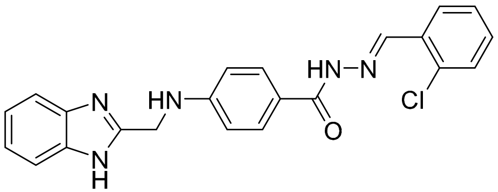

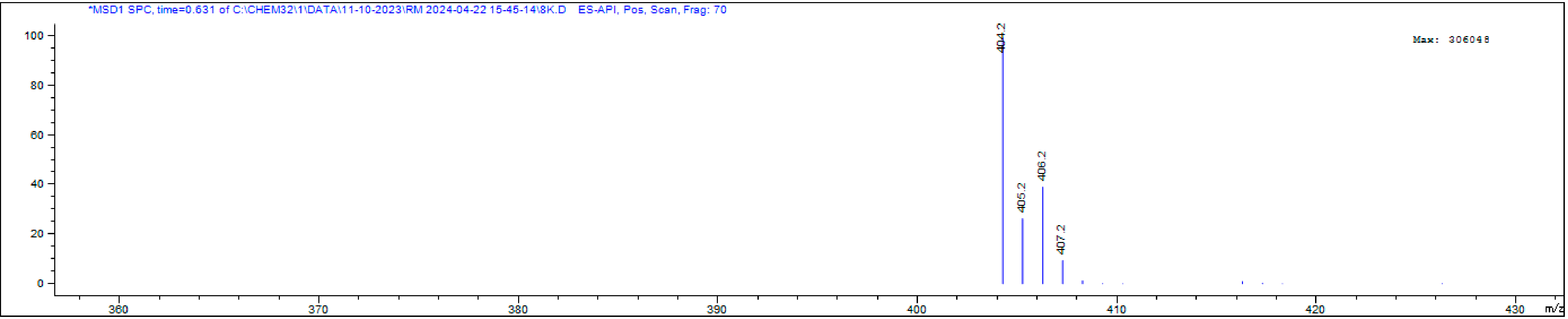

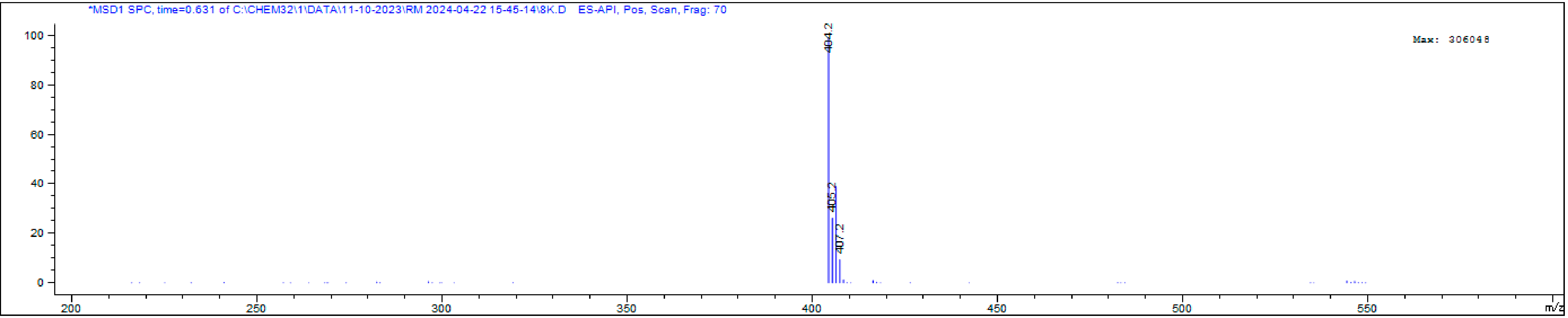

6f

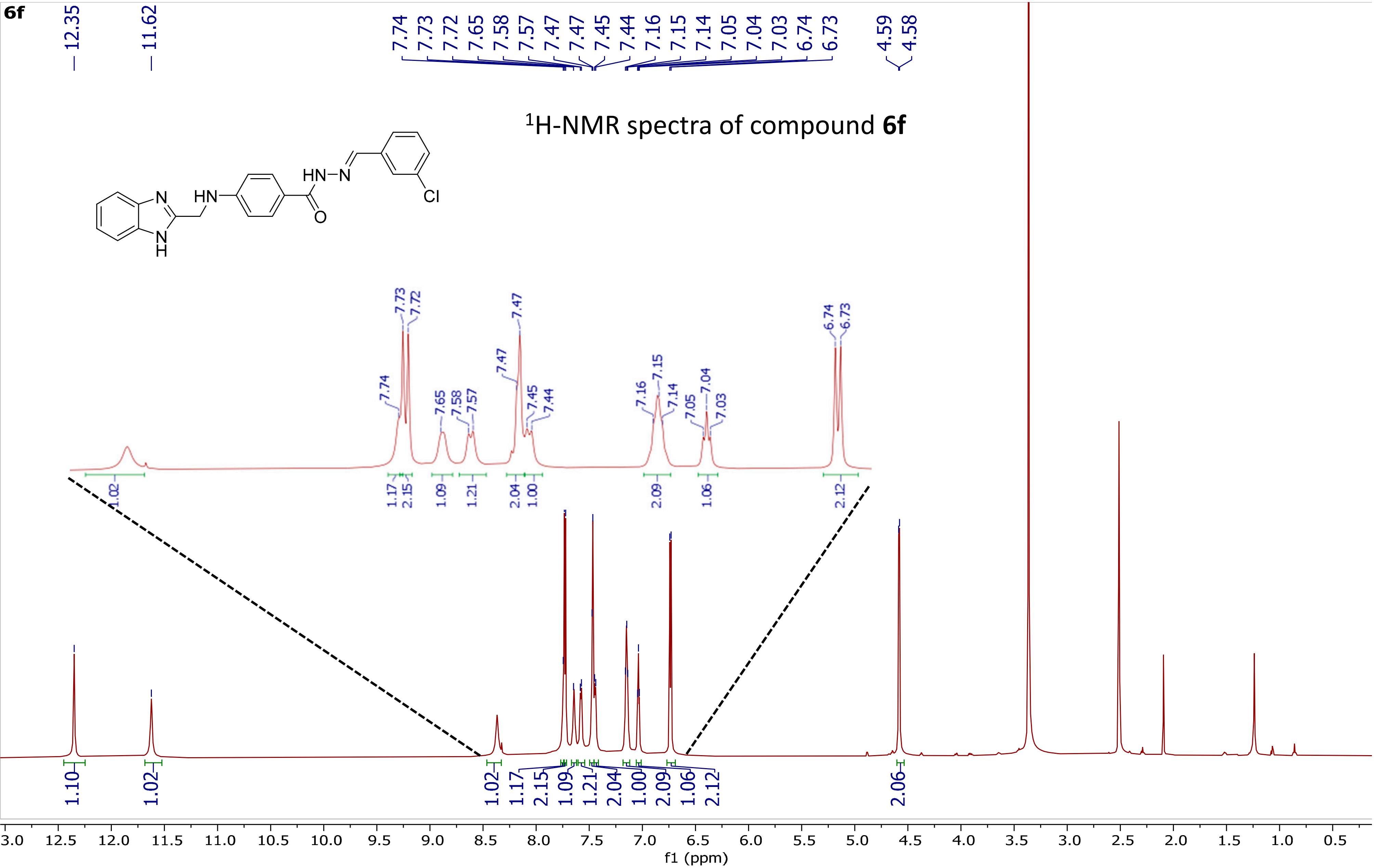

12.35
11.62
7.74
7.73
7.72
7.65
7.58
7.57
7.47
7.47
7.45
7.44
7.16
7.15
7.14
7.05
7.04
7.03
6.74
6.73
4.59
4.58
^{1}H-NMR spectra of compound **6f**
HN
N
N
N
H
HN
O
Cl
1.10
1.02
1.02
1.17
2.15
1.09
1.21
2.04
1.00
2.09
1.06
2.12
2.06
f1 (ppm)

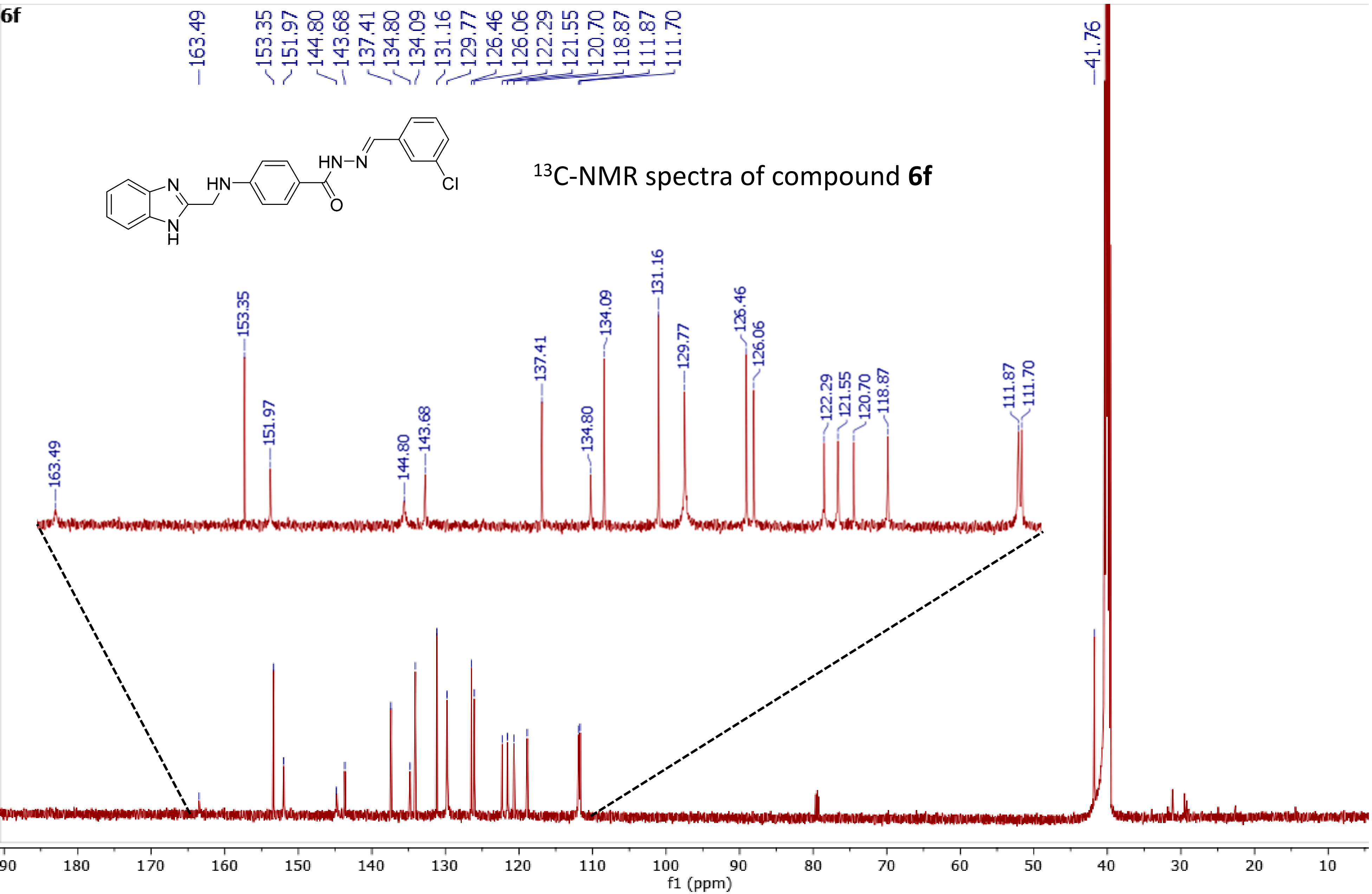
6f
13C-NMR spectra of compound 6f
163.49
153.35
151.97
144.80
143.68
137.41
134.80
134.09
131.16
129.77
126.46
126.06
122.29
121.55
120.70
118.87
111.87
111.70
41.76
f1 (ppm)

Mass spectra of compound **6f**

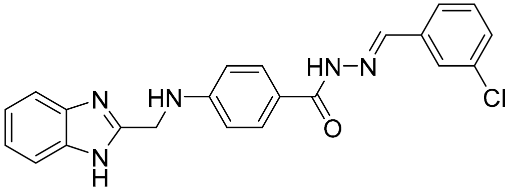

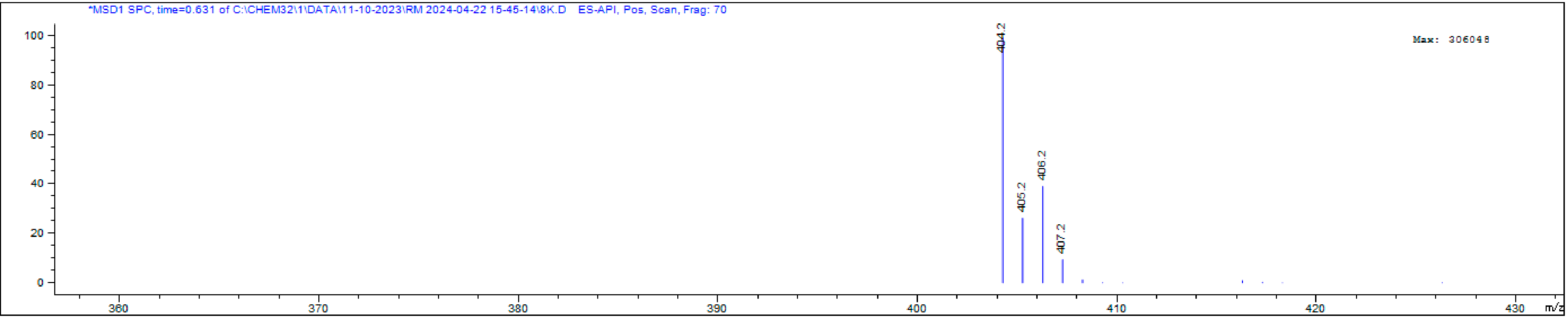

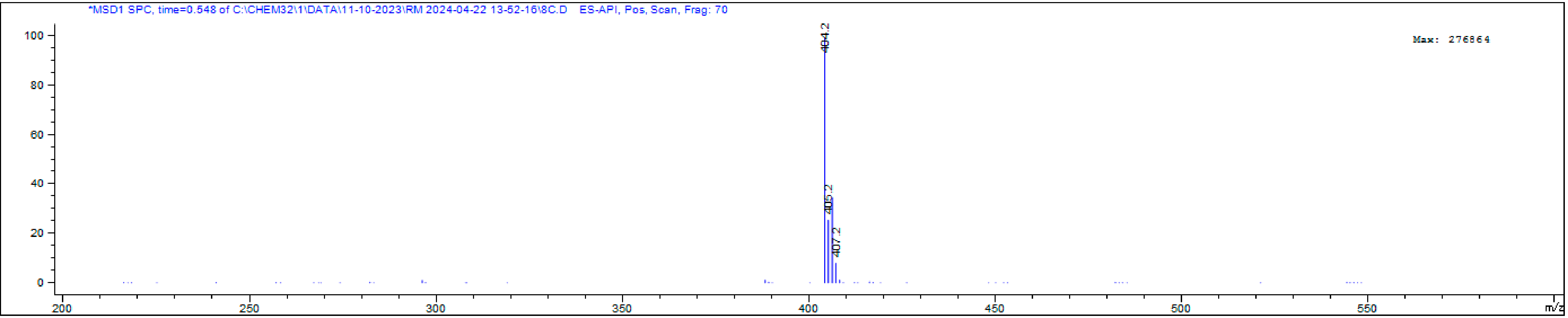

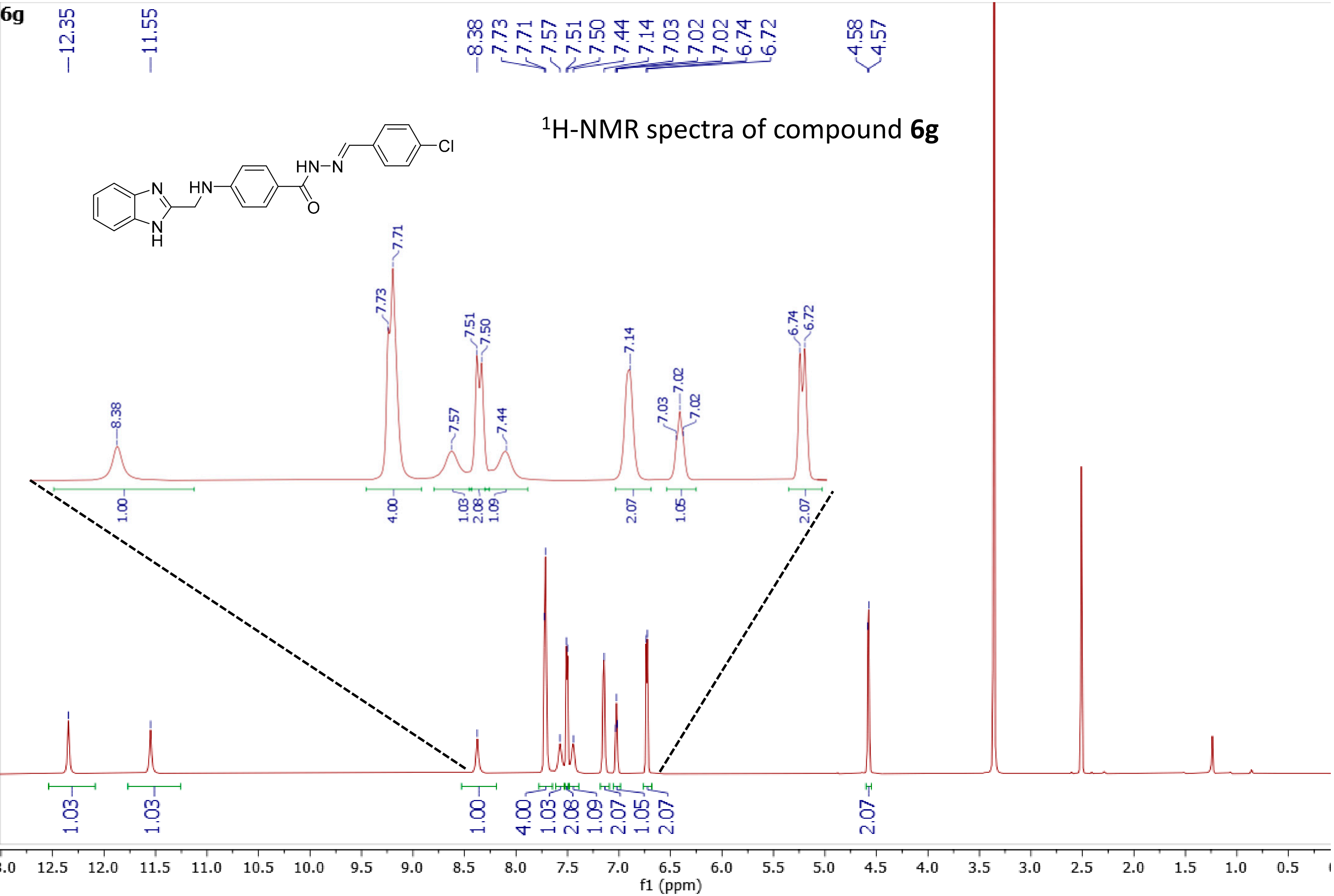

^{1}H-NMR spectra of compound **6g**

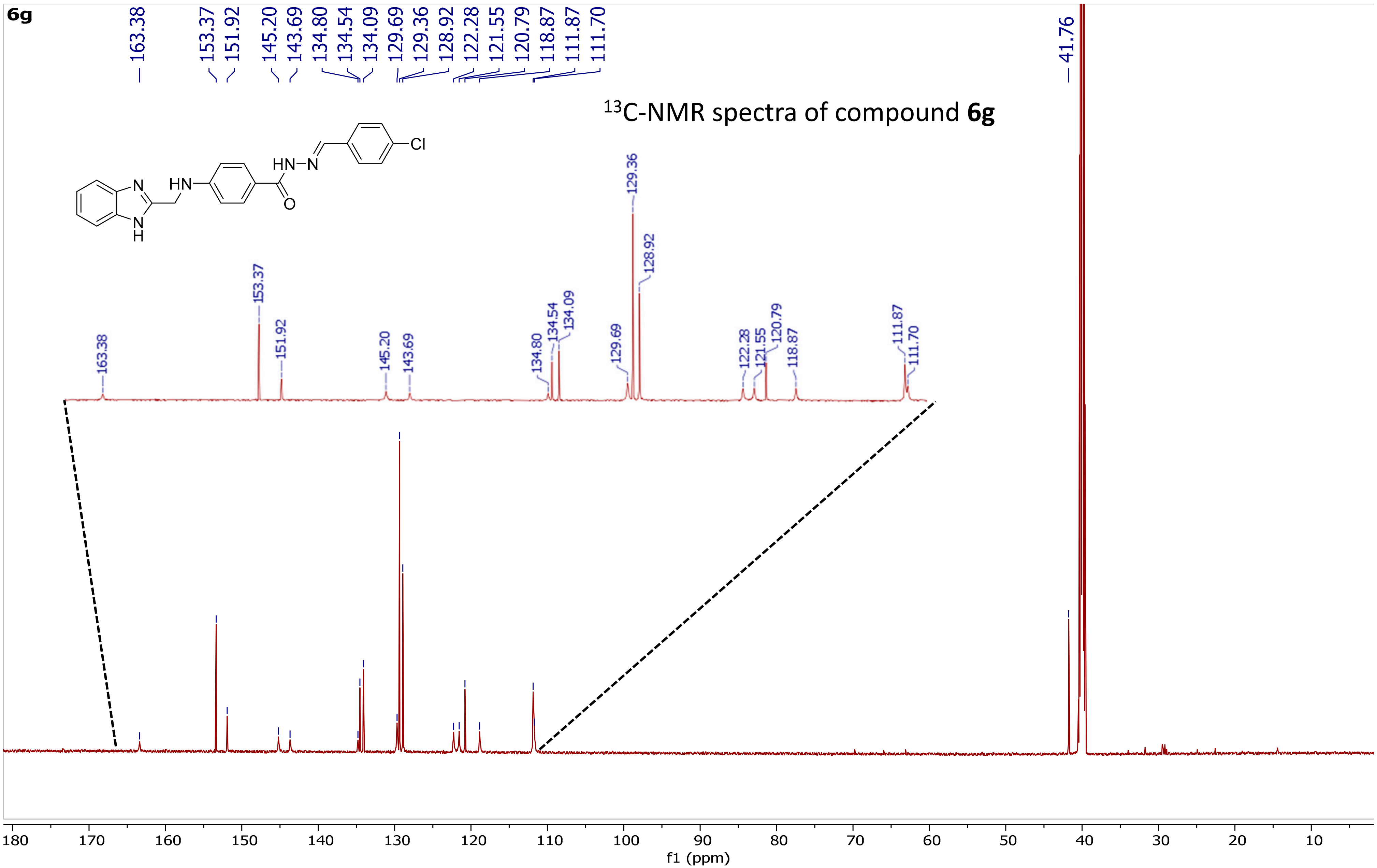
6g
163.38
153.37
151.92
145.20
143.69
134.80
134.54
134.09
129.69
129.36
128.92
122.28
121.55
120.79
118.87
111.87
111.70
41.76
13C-NMR spectra of compound 6g
HN–N
Cl
N
HN
O
N
H
180 170 160 150 140 130 120 110 100 90 80 70 60 50 40 30 20 10
f1 (ppm)

Mass spectra of compound **6g**

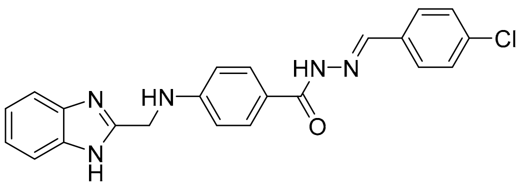

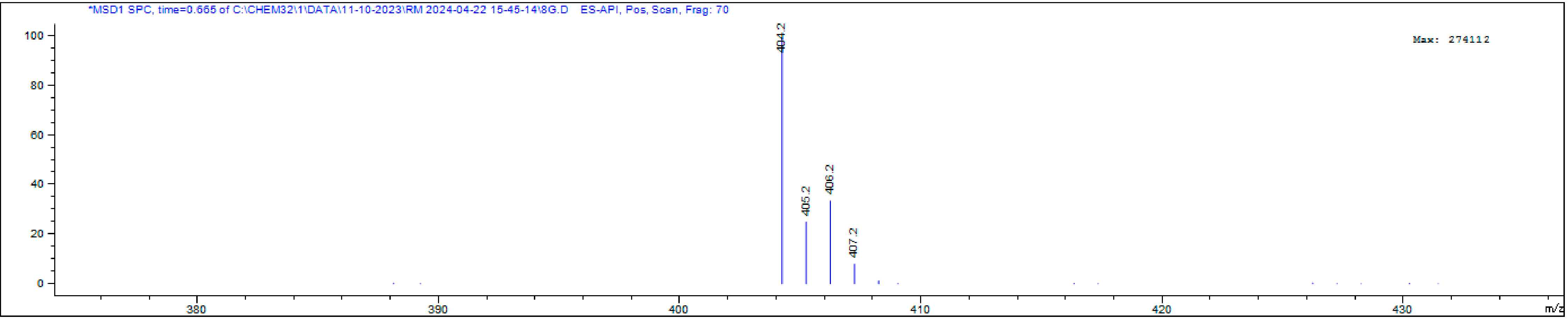

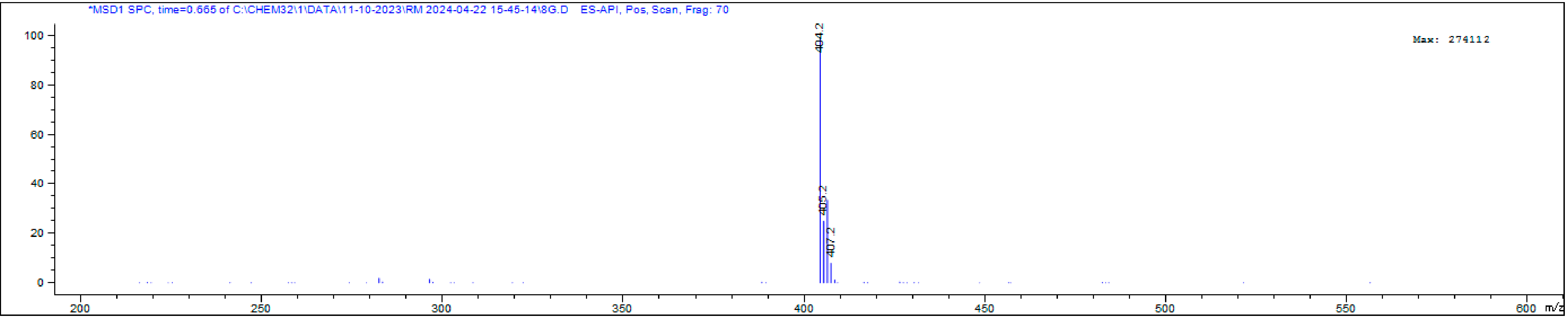

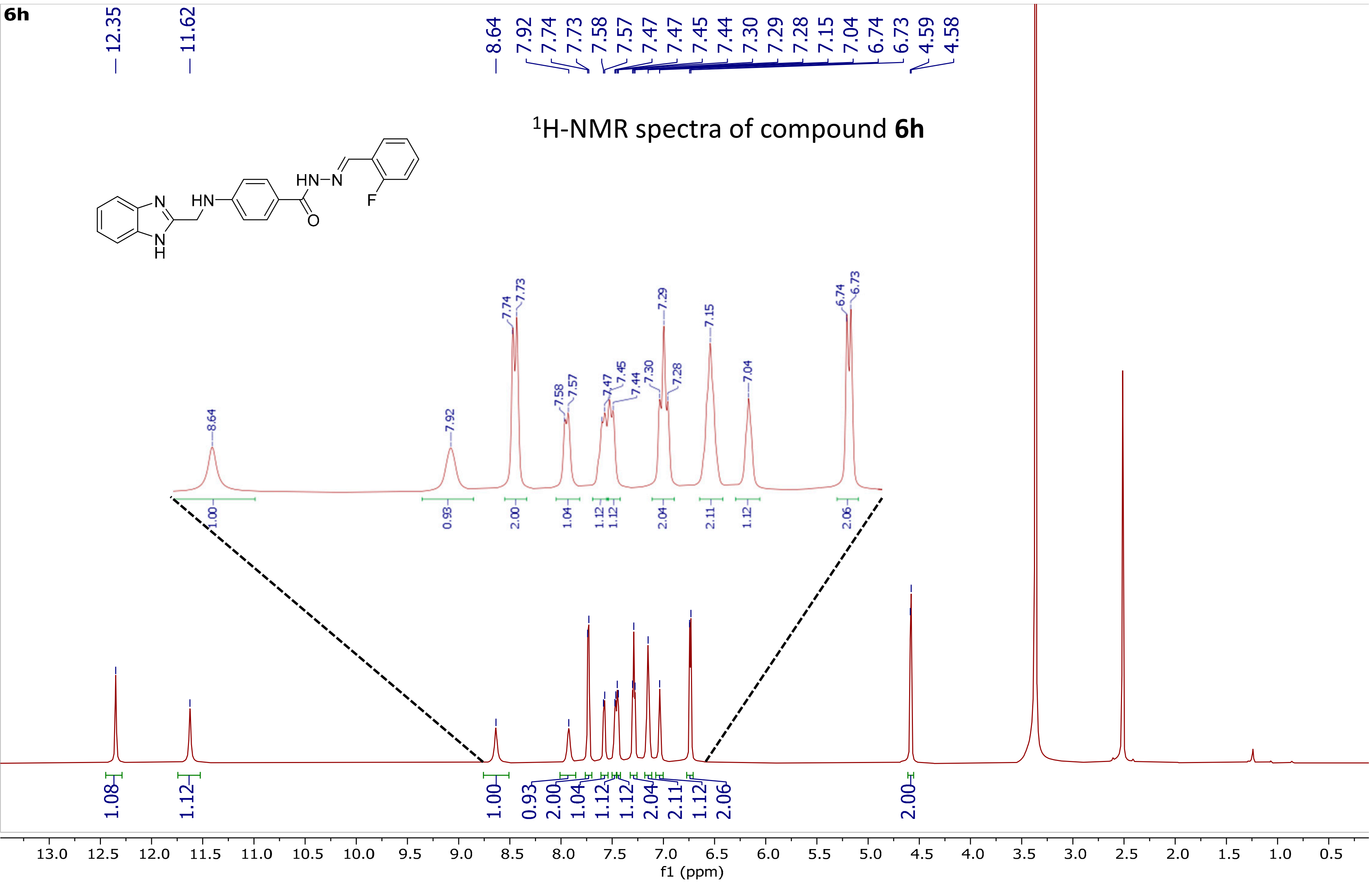
6h
1H-NMR spectra of compound 6h
HN–N
N
HN
N
H
O
F
f1 (ppm)

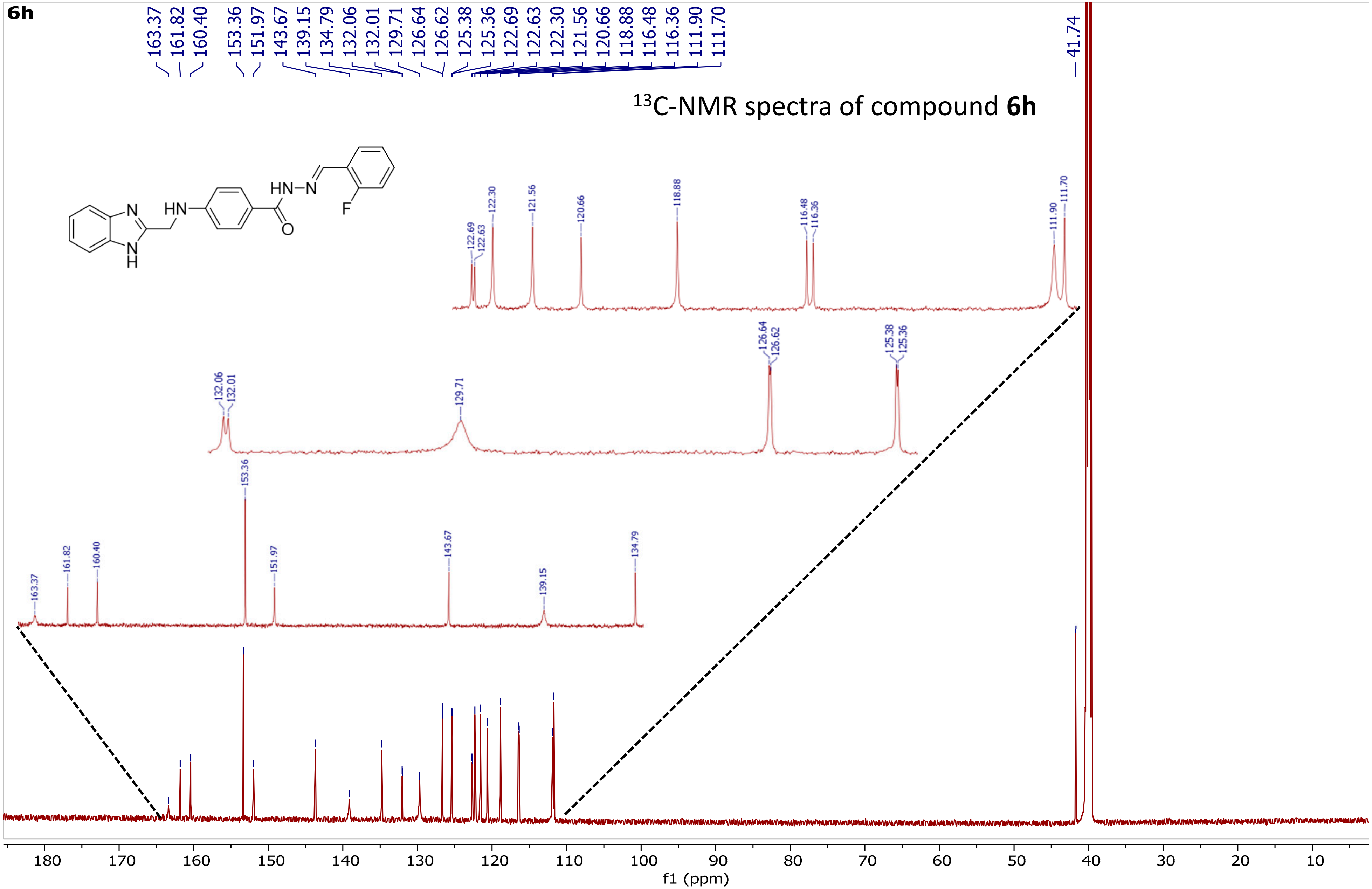
6h
13C-NMR spectra of compound 6h
163.37
161.82
160.40
153.36
151.97
143.67
139.15
134.79
132.06
132.01
129.71
126.64
126.62
125.38
125.36
122.69
122.63
122.30
121.56
120.66
118.88
116.48
116.36
111.90
111.70
41.74
HN
N
F
O
NH
H
180 170 160 150 140 130 120 110 100 90 80 70 60 50 40 30 20 10
f1 (ppm)

Mass spectra of compound **6h**

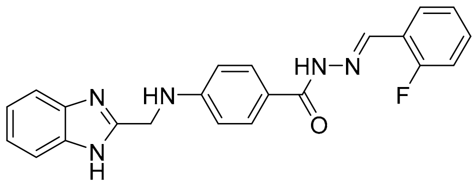

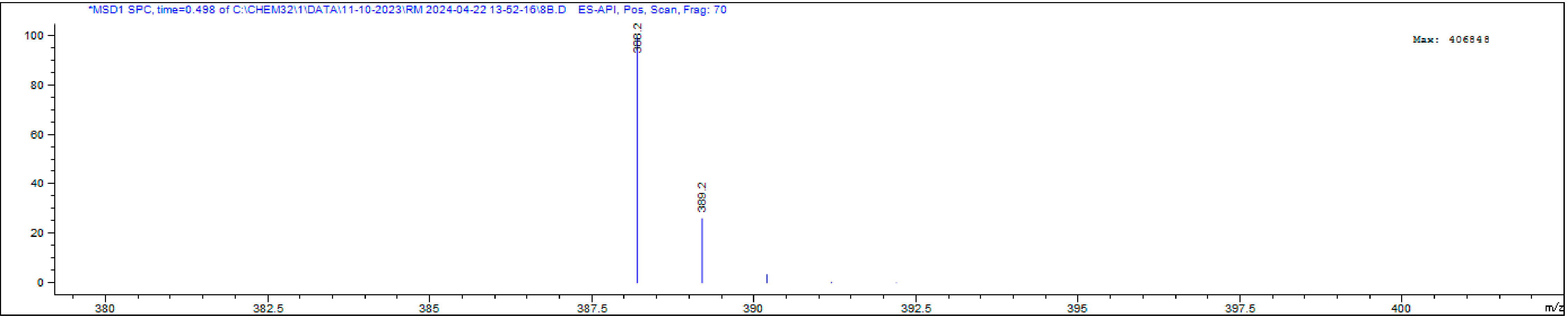

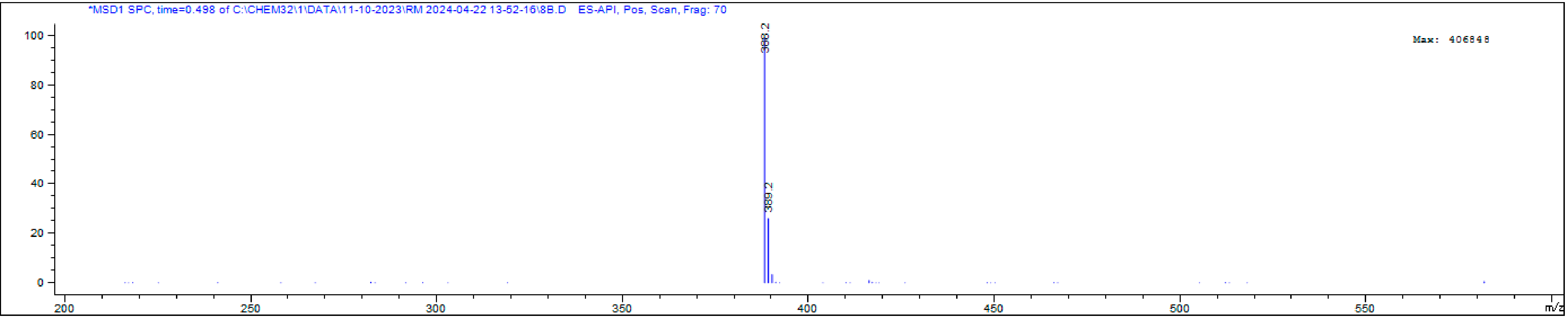

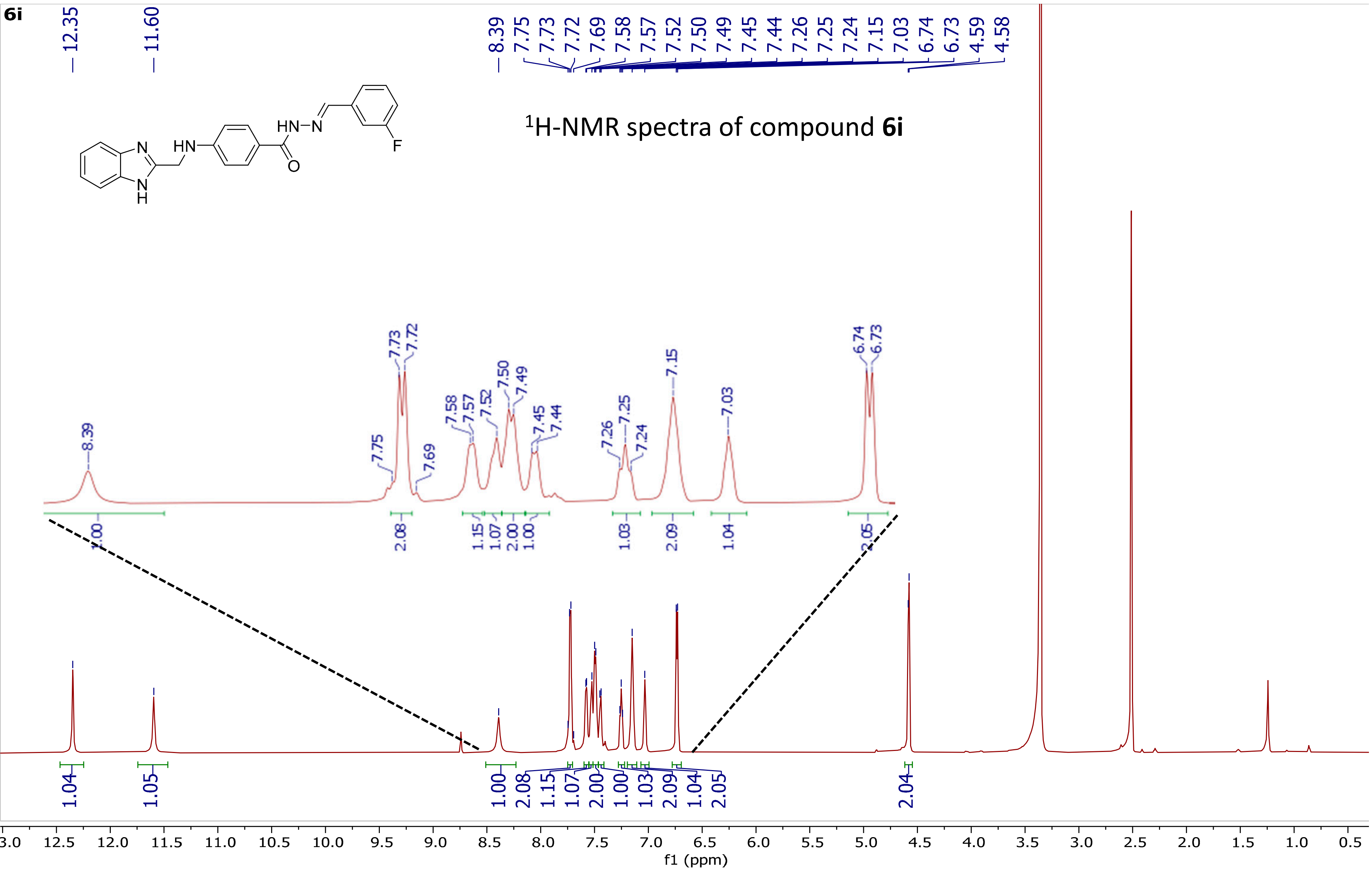

6i
1H-NMR spectra of compound 6i
12.35
11.60
8.39
7.75
7.73
7.72
7.69
7.58
7.57
7.52
7.50
7.49
7.45
7.44
7.26
7.25
7.24
7.15
7.03
6.74
6.73
4.59
4.58
HN
N
NH
HN
N
H
O
F
1.04
1.05
1.00
2.08
1.15
1.07
2.00
1.00
1.03
2.09
1.04
2.05
2.04
f1 (ppm)

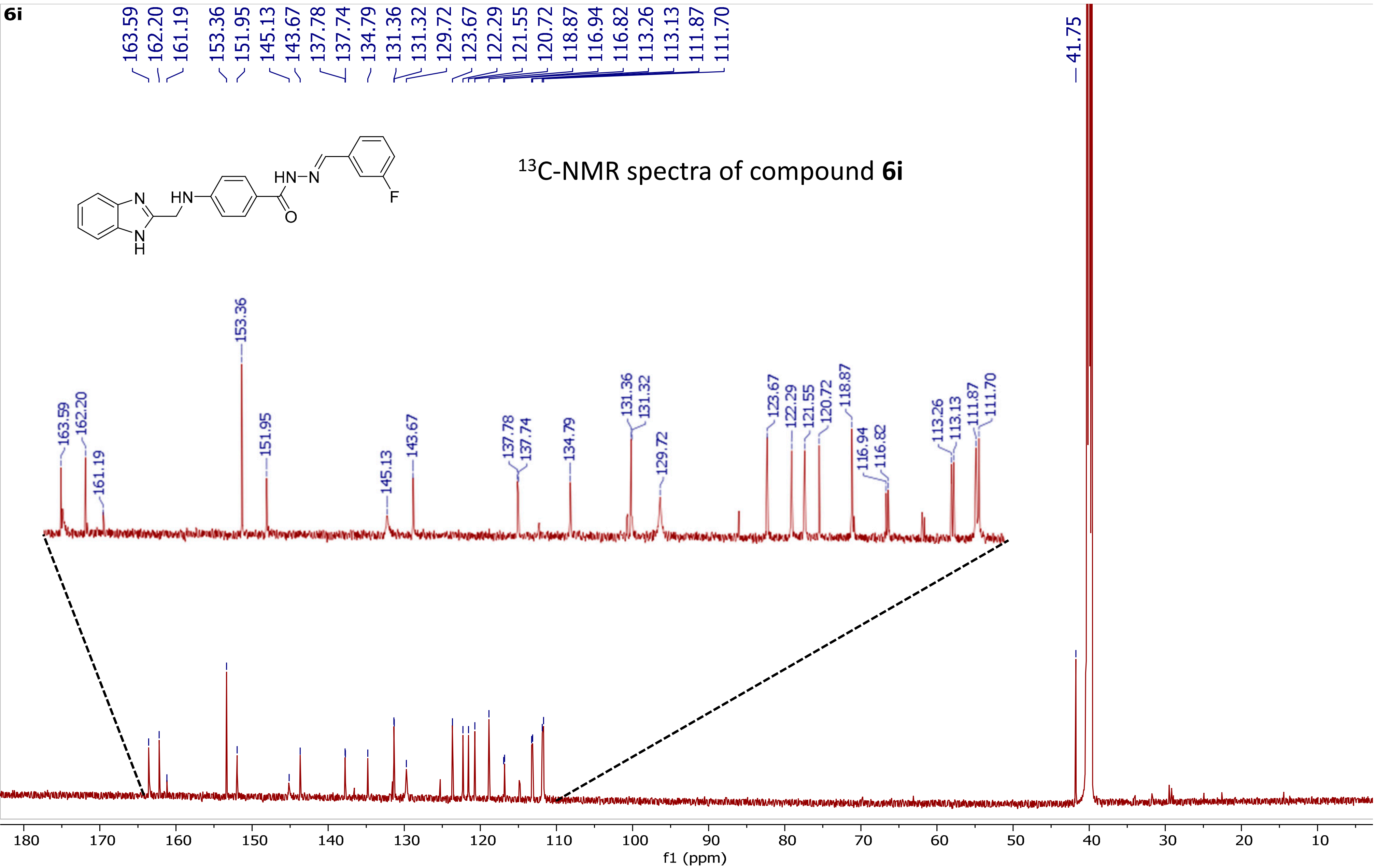
6i
13C-NMR spectra of compound 6i
163.59
162.20
161.19
153.36
151.95
145.13
143.67
137.78
137.74
134.79
131.36
131.32
129.72
123.67
122.29
121.55
120.72
118.87
116.94
116.82
113.26
113.13
111.87
111.70
41.75
f1 (ppm)

Mass spectra of compound **6i**

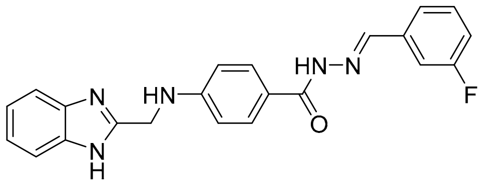

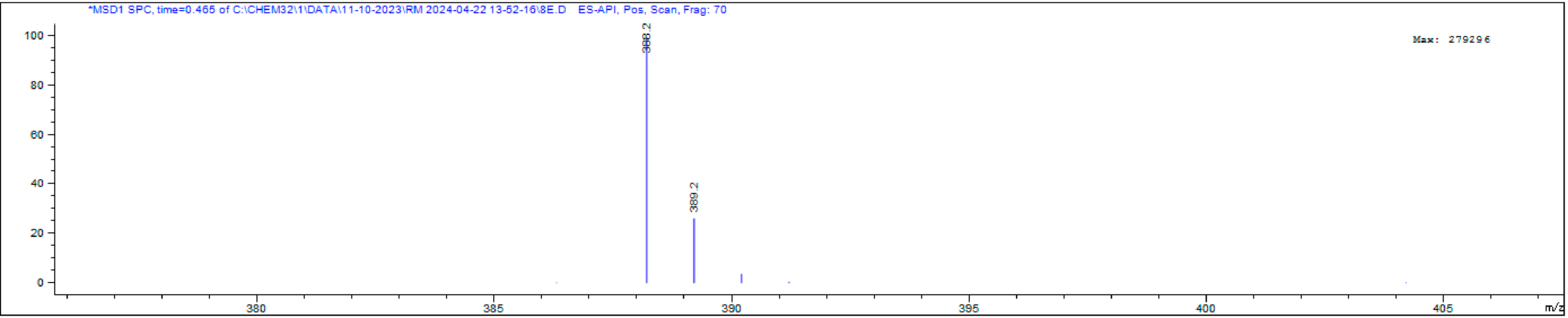

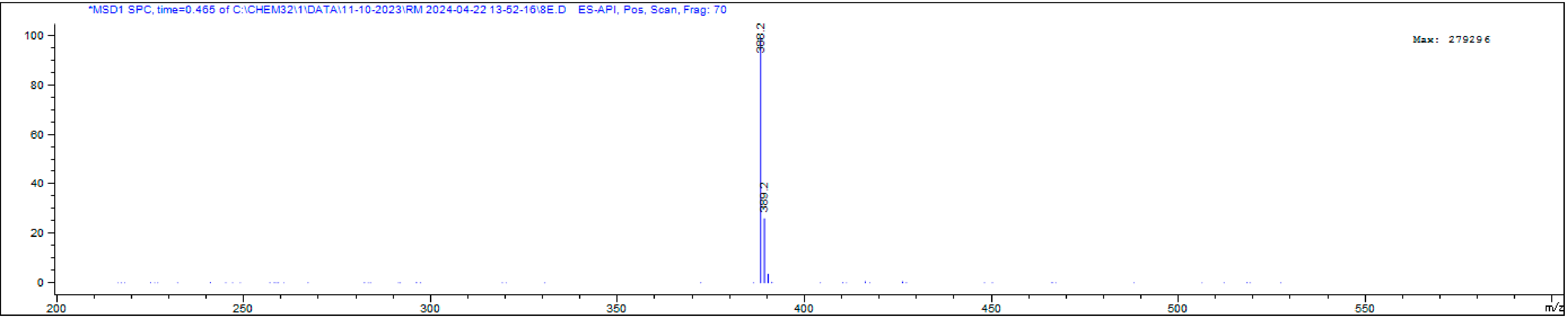

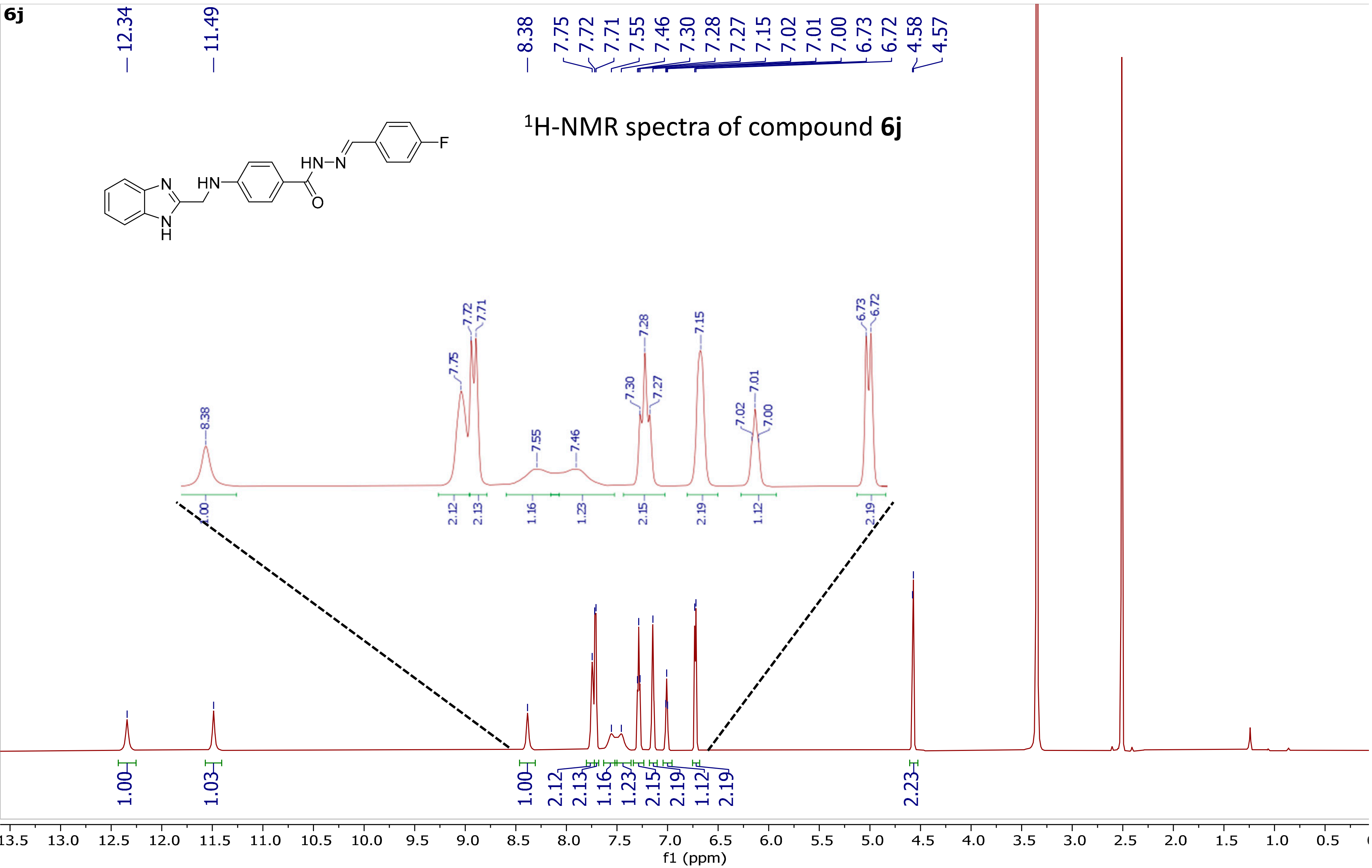
6j
12.34
11.49
8.38
7.75
7.72
7.71
7.55
7.46
7.30
7.28
7.27
7.15
7.02
7.01
7.00
6.73
6.72
4.58
4.57
^{1}H-NMR spectra of compound **6j**
HN–N
F
N
HN
N
H
O
1.00
1.03
2.12
2.13
1.16
1.23
2.15
2.19
1.12
2.19
2.23
f1 (ppm)

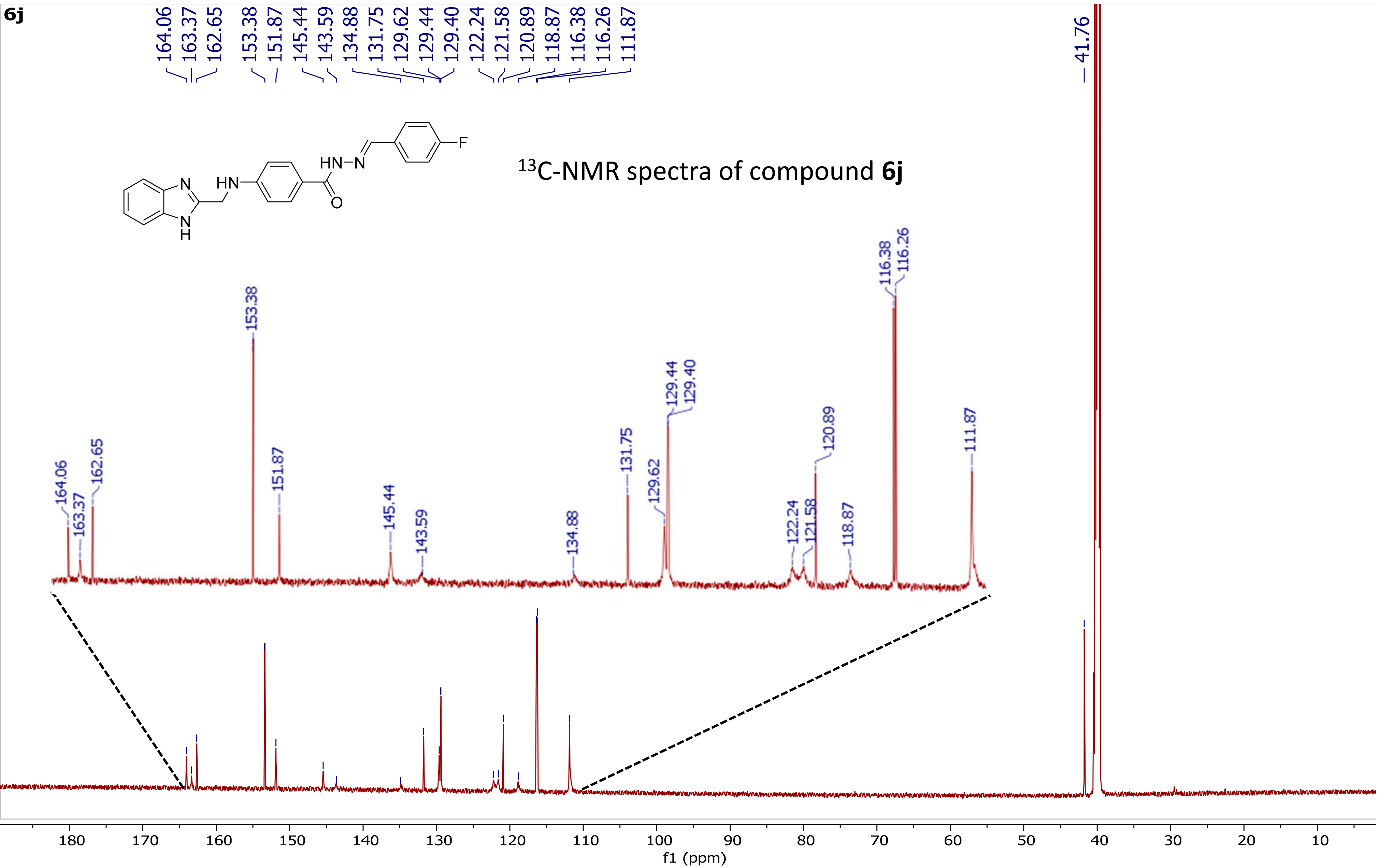

^{13}C-NMR spectra of compound **6j**

Mass spectra of compound **6j**

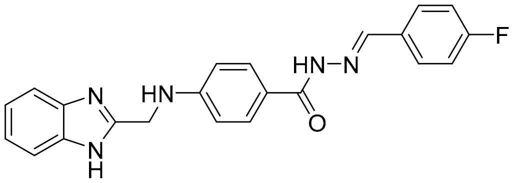

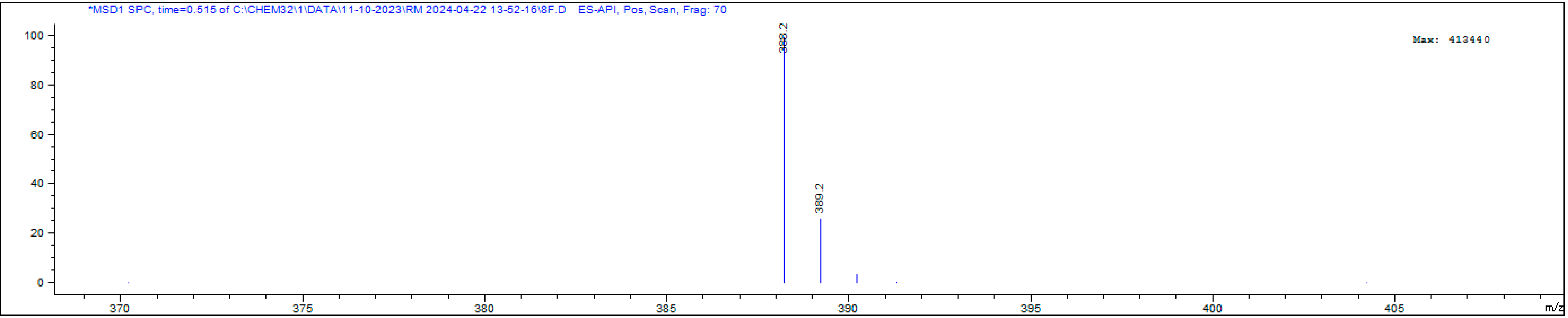

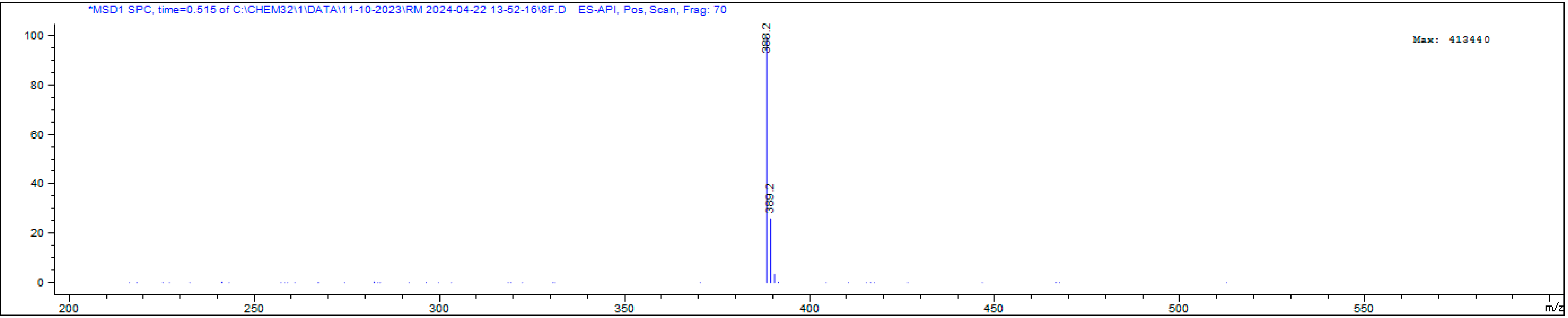

6k

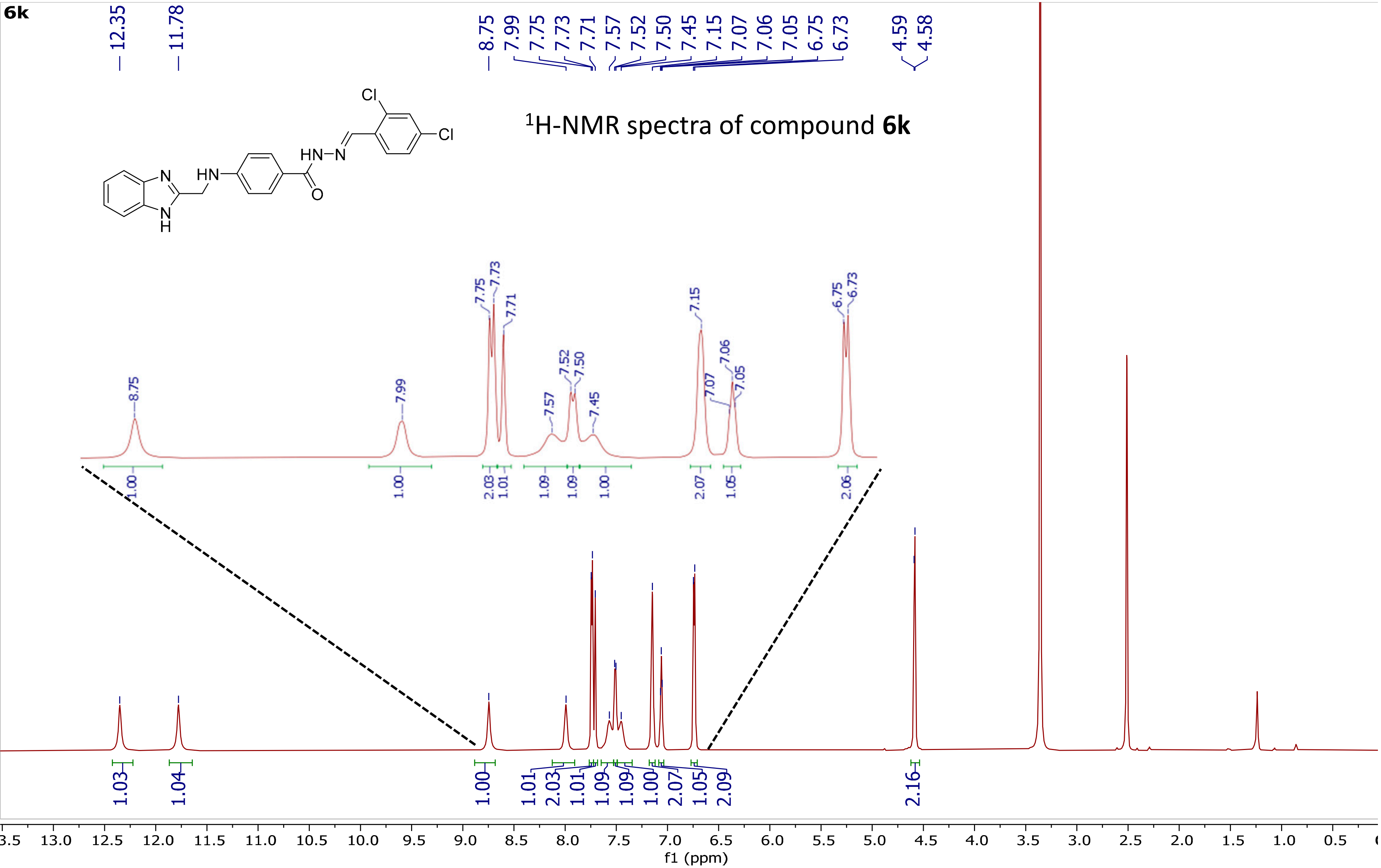

^{1}H-NMR spectra of compound **6k**

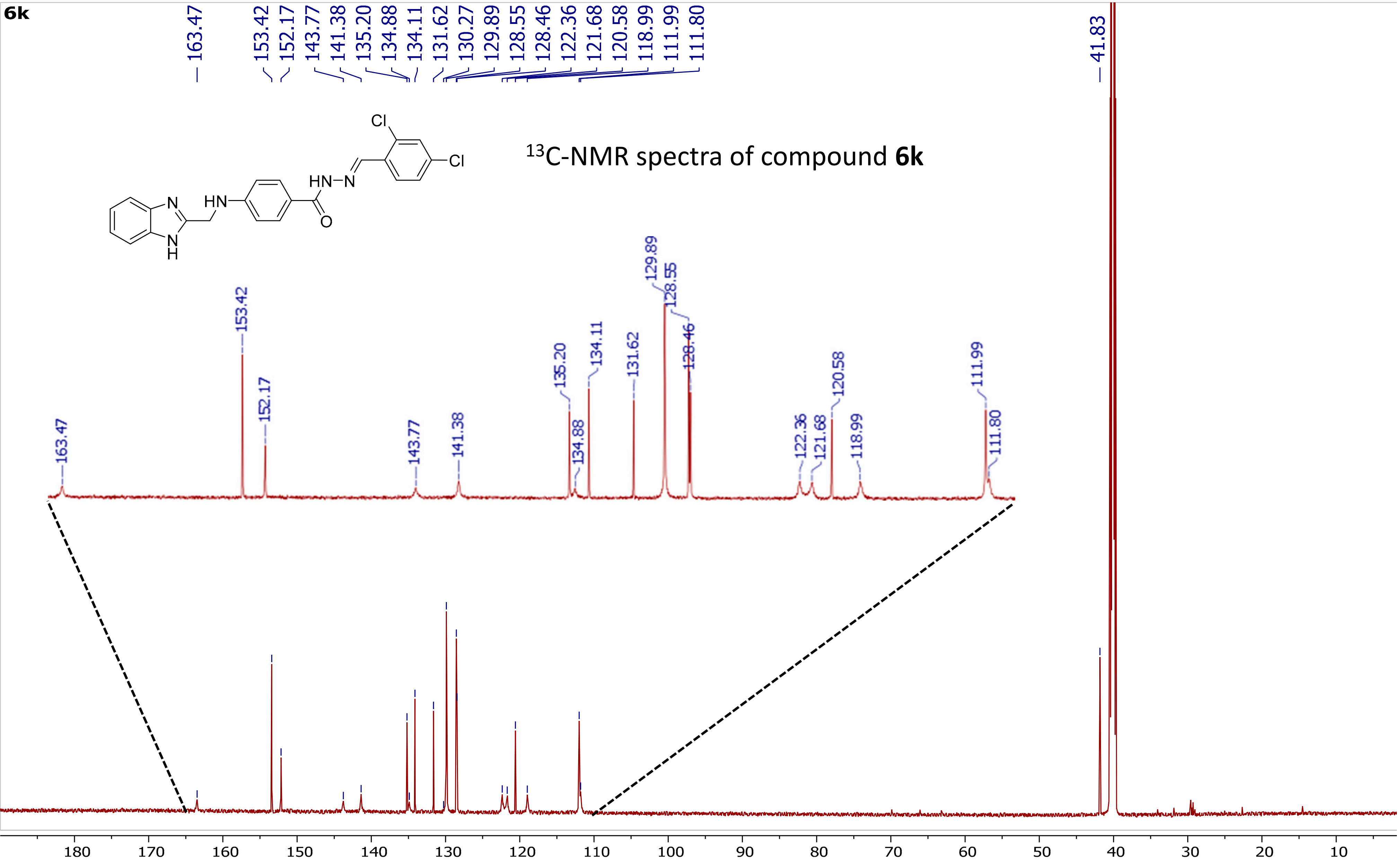
6k
163.47
153.42
152.17
143.77
141.38
135.20
134.88
134.11
131.62
130.27
129.89
128.55
128.46
122.36
121.68
120.58
118.99
111.99
111.80
41.83
Cl
Cl
HN–N
HN
N
N
H
O
^{13}C-NMR spectra of compound **6k**
180
170
160
150
140
130
120
110
100
90
80
70
60
50
40
30
20
10

Mass spectra of compound **6k**

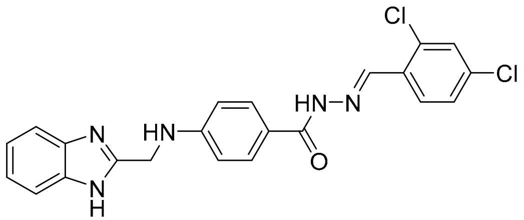

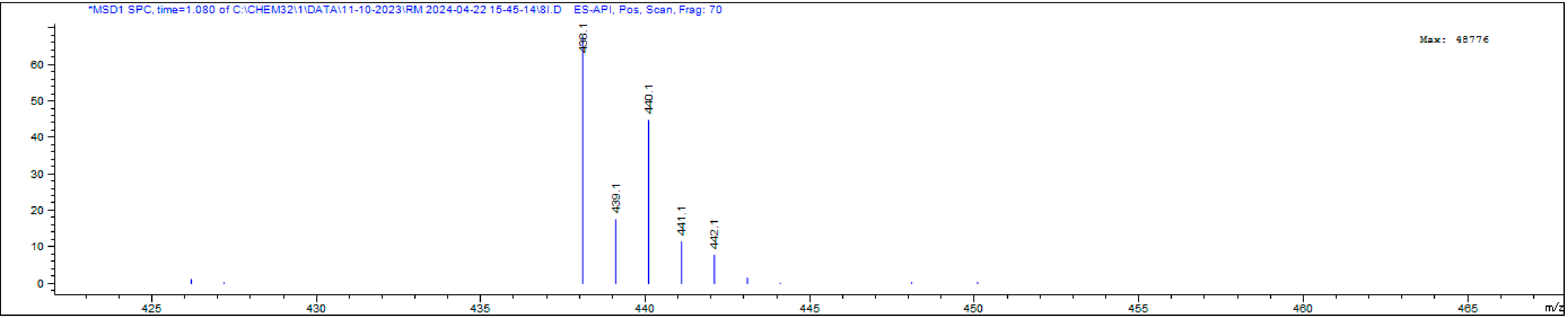

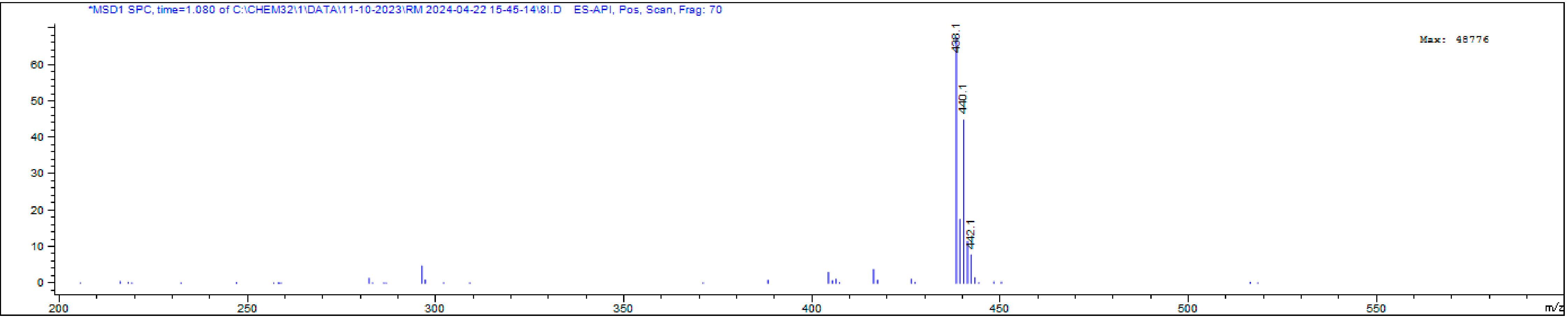

6l

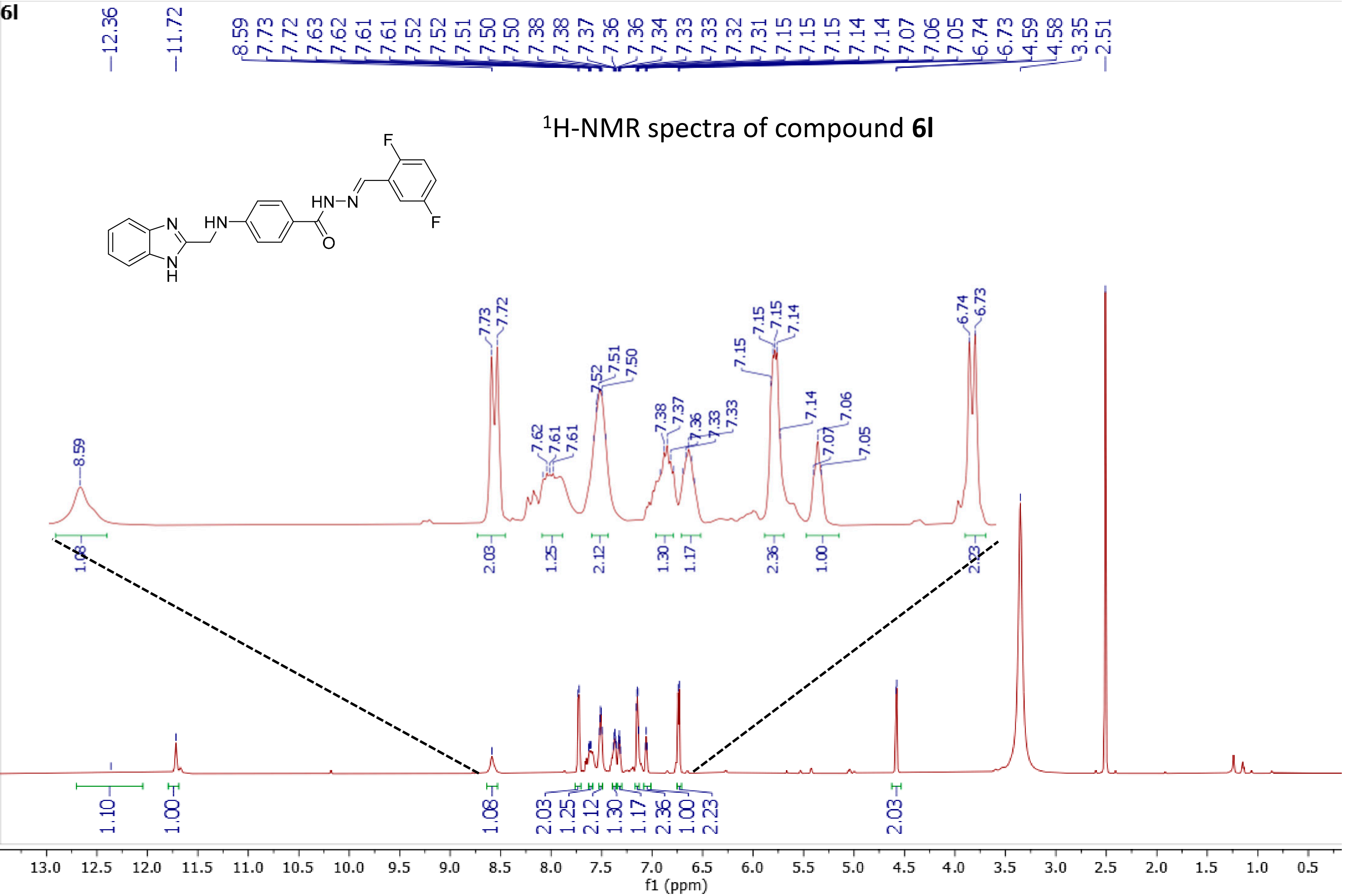

^{1}H-NMR spectra of compound **6l**

6l

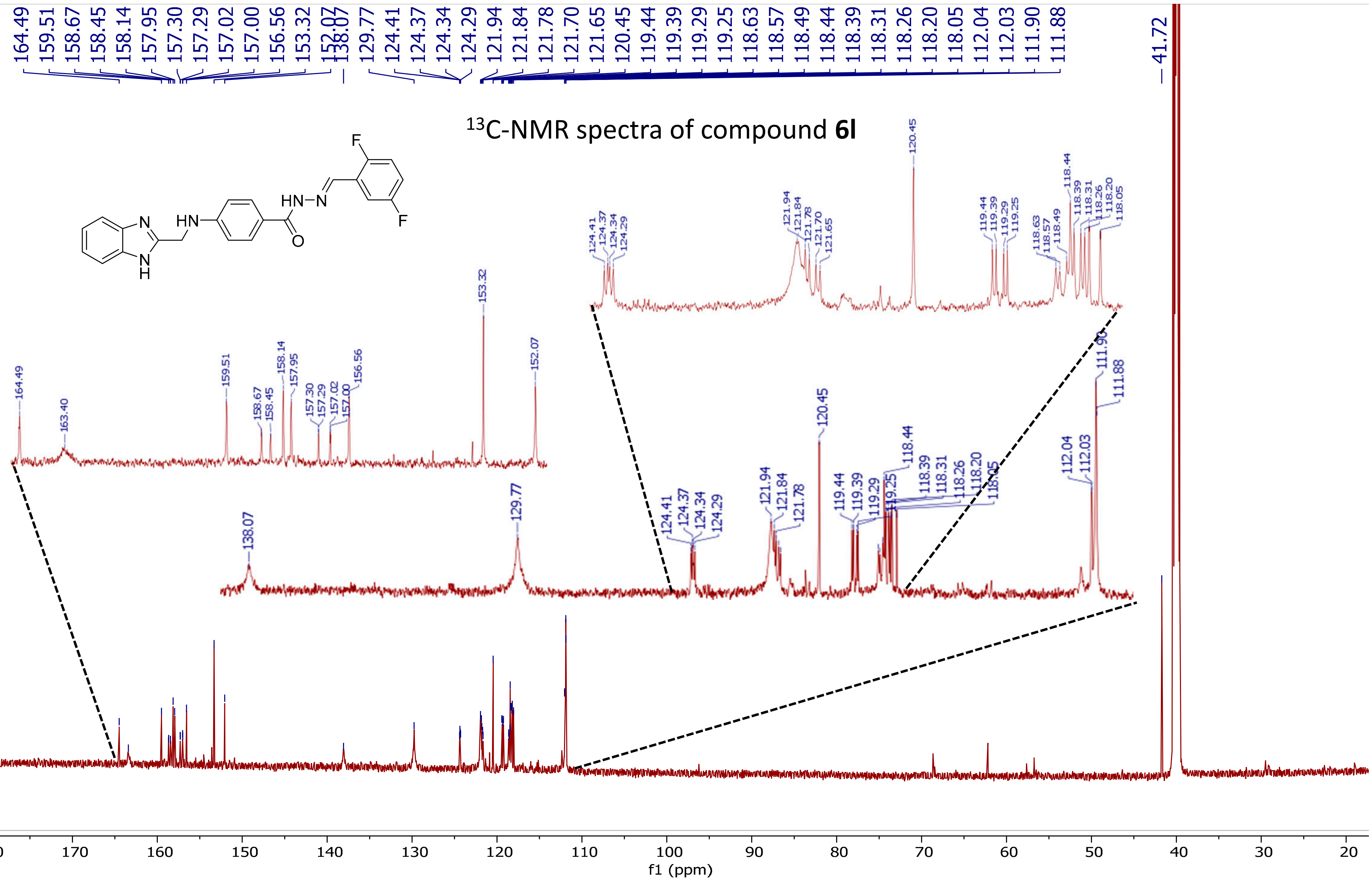

164.49 159.51 158.67 158.45 158.14 157.95 157.30 157.29 157.02 157.00 156.56 153.32 152.07 138.07 129.77 124.41 124.37 124.34 124.29 121.94 121.84 121.78 121.70 121.65 120.45 119.44 119.39 119.29 119.25 118.63 118.57 118.49 118.44 118.39 118.31 118.26 118.20 118.05 112.04 112.03 111.90 111.88
41.72
13C-NMR spectra of compound 6l
HN
N
N
H
HN
N
O
F
F
f1 (ppm)

Mass spectra of compound **6l**

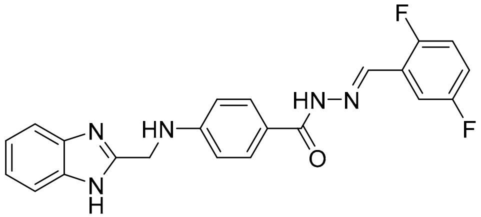

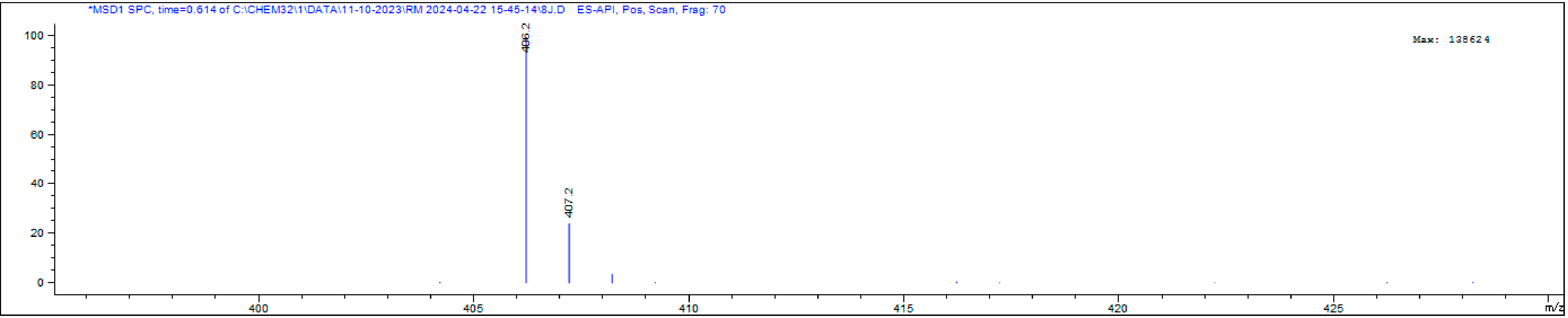

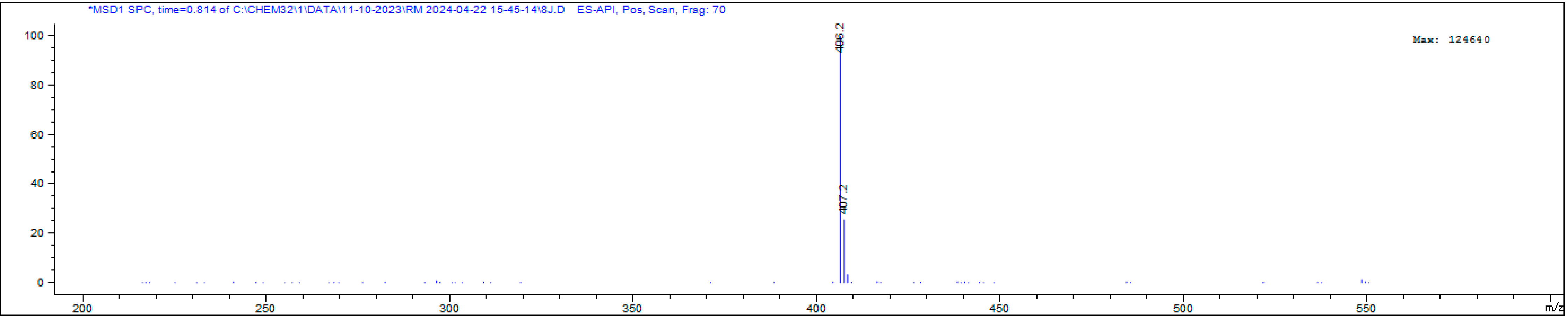

Supplement: Supplementary file 1 [file pharmaceuticals-17-00839-s001.zip › pharmaceuticals-3064363-supplementary.pdf]
